# Cation Ordering and Exsolution in Copper‐Containing Forms of the Flexible Zeolite Rho (Cu,M‐Rho; M=H, Na) and Their Consequences for CO_2_ Adsorption

**DOI:** 10.1002/chem.202101664

**Published:** 2021-08-06

**Authors:** Magdalena M. Lozinska, Sophie Jamieson, Maarten C. Verbraeken, David N. Miller, Bela E. Bode, Claire A. Murray, Stefano Brandani, Paul A. Wright

**Affiliations:** ^1^ EaStCHEM School of Chemistry University of St. Andrews Purdie Building, North Haugh St Andrews, Fife KY16 9ST UK; ^2^ School of Engineering University of Edinburgh The King's Buildings Robert Stevenson Road Edinburgh EH9 3FB UK; ^3^ Diamond Light Source Ltd. Harwell Science and Innovation Campus Didcot, Oxfordshire OX11 0DE UK

**Keywords:** copper zeolites, CO_2_ adsorption, CO_2_ separation, exsolution, zeolite Rho

## Abstract

The flexibility of the zeolite Rho framework offers great potential for tunable molecular sieving. The fully copper‐exchanged form of Rho and mixed Cu,H‐ and Cu,Na‐forms have been prepared. EPR spectroscopy reveals that Cu^2+^ ions are present in the dehydrated forms and Rietveld refinement shows these prefer S6R sites, away from the *d8r* windows that control diffusion. Fully exchanged Cu‐Rho remains in an open form upon dehydration, the *d8r* windows remain nearly circular and the occupancy of window sites is low, so that it adsorbs CO_2_ rapidly at room temperature. Breakthrough tests with 10 % CO_2_/40 % CH_4_ mixtures show that Cu_4.9_‐Rho is able to produce pure methane, albeit with a relatively low capacity at this p_CO2_ due to the weak interaction of CO_2_ with Cu cations. This is in strong contrast to Na‐Rho, where cations in narrow elliptical window sites enable CO_2_ to be adsorbed with high selectivity and uptake but too slowly to enable the production of pure methane in similar breakthrough experiments. A series of Cu,Na‐Rho materials was prepared to improve uptake and selectivity compared to Cu‐Rho, and kinetics compared to Na‐Rho. Remarkably, Cu,Na‐Rho with >2 Cu cations per unit cell exhibited exsolution, due to the preference of Na cations for narrow S8R sites in distorted Rho and of Cu cations for S6R sites in the centric, open form of Rho. The exsolved Cu,Na‐Rho showed improved performance in CO_2_/CH_4_ breakthrough tests, producing pure CH_4_ with improved uptake and CO_2_/CH_4_ selectivity compared to that of Cu_4.9_‐Rho.

## Graphical abstract

**Copper cations** favour 6‐membered ring (6R) sites in dehydrated zeolite Rho, away from 8R windows that allow adsorption into *lta* cages. Exchange of Cu cations into Na‐Rho, in which Na cations occupy 8R gating sites between the *lta* cages, improves CO_2_ uptake kinetics, while retaining some Na cations enhances CO_2_/CH_4_ selectivity. Upon dehydration, Cu,Na‐Rho shows exsolution behaviour, because its framework can adopt distorted and undistorted forms that provide favourable coordination sites for Na and Cu cations, respectively.

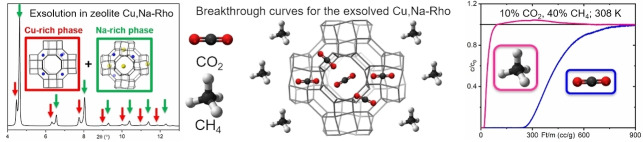

## Introduction

Zeolites find widespread use as adsorbents in a range of commercially‐important gas separations involving small molecules, including air separation (where N_2_/O_2_ selectivity is required) and hydrogen purification (CO_2_/H_2_).[^1^, ^2^, ^3^] Furthermore, advanced materials and chemical engineering research continues to drive improved performance in these and similar applications,^[4]^ and also in CO_2_ adsorption in natural gas and biogas upgrading (CO_2_/CH_4_)[^5^, ^6^] and carbon capture from power plant and industrial emissions (CO_2_/N_2_ and CO_2_/CO,H_2_).[^7^, ^8^]

The performance of zeolites in gas separation relates directly to their high chemical and thermal stability and also to their structural features: high internal surface area accessible via well‐defined pores and the presence of extra‐framework cations. These cations affect their adsorption properties in a number of ways. First, the direct cation‐adsorbate interaction enables molecules to be differentiated based on their dipole moment or polarizability – cationic zeolites can separate N_2_ from O_2_ due to its higher polarizability, for example.[^1^, ^2^] Extra‐framework cations can also control the effective pore size, if they are located close to windows, as shown by the increasing pore size of K‐, Na‐ and Ca‐forms of zeolite Linde A (known as 3A, 4A, and 5A respectively). There is also strong evidence that cations in single eight‐membered ring (S8R) sites (8R refers to the size of the ring, which contains 8 tetrahedral Si or Al atoms and 8 O atoms) can exert trapdoor behaviour, where the cation must move to allow passage of adsorbates, leading to selectivity on the basis of the strength of cation – molecule interactions.[^7^, ^9^, ^10^, ^11^, ^12^, ^13^, ^14^, ^15^]

The extra‐framework cation composition can be modified by aqueous ion exchange. Many studies have investigated the adsorption behaviour as the cation type (and charge) is varied, typically examining the alkali and alkaline earth metal cations. For example, the adsorption of N_2_ and CO_2_ on alkali metal cation forms of the commercially‐important zeolites A and X has been compared.[^16^, ^17^, ^18^, ^19^] Most such studies have concentrated on single cation forms of zeolites, which allows for straightforward rationalisation and computational modelling, but there are examples where mixed cation forms of zeolites have important advantages in industrial applications. For example, whereas fully Li‐exchanged forms of chabazite have excellent properties for N_2_/O_2_ separation, the high lithium content compromises their structural stability, due to the strong interactions of Li cations with O atoms of the framework, and mixed Li/Na forms of chabazite have improved stability while retaining high separation.[^20^, ^21^] In these cases, site ordering of cations over different framework sites is observed, and therefore determination of the resulting distribution is essential to understand the properties. In Li,Na‐chabazite, for example, Li cations favour the smaller single six‐membered ring (S6R) sites whereas the Na cations prefer the larger 8R sites, due to their different cationic radii (Li^+^, 0.76 Å; Na^+^, 1.02 Å).[^20^, ^21^]

Some zeolites possess flexible frameworks, which, when dehydrated, adapt to achieve optimal coordination with the extra‐framework cations. Zeolite Rho is the archetypal flexible zeolite, but other zeolites have been found to exhibit similar types of behaviour.[^9^, ^22^, ^23^, ^24^, ^25^, ^26^, ^27^, ^28^] Varying the cations can change the framework configuration of the ‘activated’ adsorbent and thereby the effective pore size, particularly for molecules that interact weakly with cations, such as O_2_ and N_2_. Additionally, as the framework distorts, the geometry of cation sites changes, so that cation coordination with framework O atoms, and therefore cation site preferences, are likely to change.

**Figure 1:**
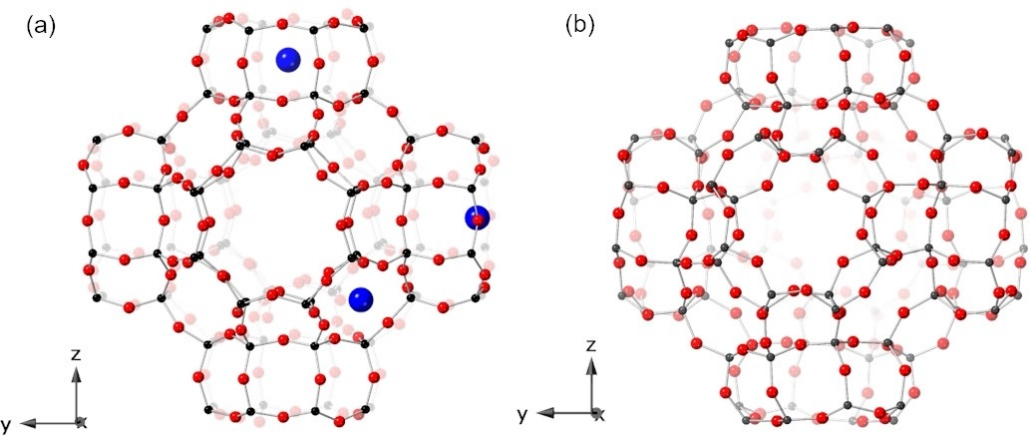
Two symmetries of zeolite Rho framework: (a) $Im\bar{3}m$ space group including three main cation sites: single eight‐membered ring (S8R), double eight‐membered ring (D8R) and single six‐membered ring (S6R) and (b) $I\bar{4}3m$ space group. Oxygen atoms=red spheres, T atoms (Si or Al)=grey spheres and cations=blue spheres.

The CO_2_ adsorption properties of univalent cation forms of zeolite Rho have been studied extensively.[^9^, ^22^, ^23^, ^29^] Rho is a promising zeolite for adsorption applications because of its large, three‐dimensionally connected pore volume, which comprises two identical, interpenetrated, but unconnected pore systems comprising *lta* cages connected by *d8r* windows, in which all of the internal space is accessible to small molecules (Figure 1a). When in the hydrated form, zeolite Rho adopts $Im\bar{3}m\;$ symmetry, with *a*=15.0352(2) Å,^[22]^ but upon dehydration the framework can distort as the *d8r* unit twists, to give the acentric variant, space group $I\bar{4}3m$ (Figure 1b).

The Rho framework offers three main cation sites: single eight‐membered ring (S8R) and double eight‐membered ring (D8R) sites in the *d8r*s connecting *lta* cages, and single six‐membered ring (S6R) sites in the *lta* cages (Figure 1a). In the proton form, zeolite H‐Rho(3.9), the structure retains $Im\bar{3}m\;$ symmetry, even when dehydrated, but in Na_9.8_‐Rho (9.8 cations per unit cell) the structure distorts to $I\bar{4}3m$ , with unit cell *a* parameters of 14.3771(2) Å.^[22]^ This enables metal cations to achieve closer coordination with framework O atoms and therefore more favourable coulombic interaction (Figure 1b). In Na‐Rho, Na cations show a preference for the distorted S8R sites, with S6R sites being filled only when the *d8r* windows are occupied by at least one Na cation (in S8R sites). The cations in S8R sites block the windows and must move to allow sorbate uptake via trapdoor mechanism. This imparts a very high CO_2_/CH_4_ selectivity, even at high CH_4_ pressure, but the diffusion in Na‐Rho is very slow.[^22^, ^30^]

In this study we aimed to improve transport properties of Na‐Rho by ion exchange with a divalent cation likely to prefer S6R sites. This should reduce the number of cations in total, due to charge balance considerations, and also decrease the likelihood that cations occupy sites in the *d8r* window. Cu^2+^ was chosen as a divalent cation likely to show a preference for S6R sites. Much is known of the siting of Cu^2+^ in high Si/Al ratio, small pore zeolites because of the catalytic application of such materials in the selective catalytic reduction of NO by NH_3_ (SCR) in diesel engine exhausts. The structural study of the Cu‐form of the high Si/Al form of zeolite A, performed as part of an examination of the remarkable SCR activity of this material, is particularly relevant in our case, because the Cu^2+^ is found to occupy S6R sites within the *lta* cage, which is also found in the zeolite Rho structure.^[31]^ (Notably, the Cu‐form of high silica zeolite Rho (with Si/Al up to 12.5) shows promising SCR activity.^[32]^) Furthermore, measurements of the O_2_, N_2_ and Ar uptake on Cu_4.9_‐Rho indicate very low O_2_/Ar and O_2_/N_2_ selectivity and very high O_2_ diffusion rates similar to those of H‐Rho,^[4]^ which suggests Cu cations are primarily located away from *d8r* windows.

Here, we have prepared Cu,H‐ and Cu,Na‐Rho materials by aqueous ion exchange and deammoniation. Copper cations are found to prefer S6R sites strongly in the dehydrated forms of these materials. This favours open structures, and leads to exsolution phenomena in some Cu,Na‐Rho compositions. Determination of the kinetics and selectivity of CO_2_ adsorption via zero length column and breakthrough curve measurements indicate that a mixed cation Cu_3.4_Na_3_‐Rho has an improved combination of uptake, kinetics and CO_2_/CH_4_ selectivity over end‐member Cu‐ or Na‐Rho compositions.

## Results and Discussion

Cu,H‐Rho samples were prepared with compositions Cu_0.9_H_8.0_‐Rho, Cu_2.1_H_5.6_‐Rho, Cu_3.0_H_3.8_‐Rho and Cu_4.9_‐Rho as described in the Experimental section and Supporting Information. The diffraction patterns were measured before and after calcination (see Figure S1 in Supporting Information) and no extra peaks corresponding to extra copper‐containing phases were observed. The gradual decrease in intensity of the {110} peak indicated greater exchange of NH_4_^+^ cations for Cu^2+^ cations in each consecutive sample.

**Figure 2:**
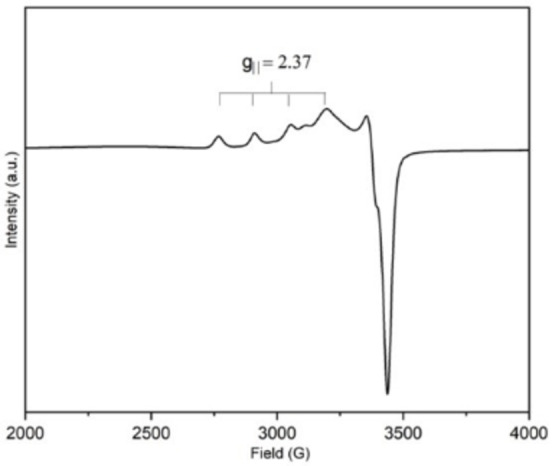
EPR spectrum of dehydrated Cu_0.9_H_8.0_‐Rho sample measured at 295 K; the g|| region is highlighted.

**Figure 3:**
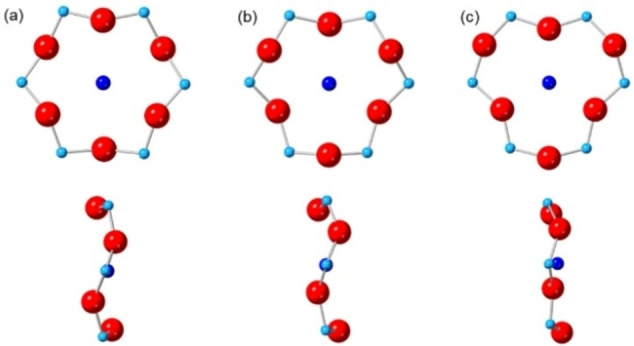
Position of Cu^2+^ cations in S6R sites in (a) Cu_0.9_H_8.0_‐Rho, (b) Cu_4.9_‐Rho and (c) Cu_1.0_Na_7.8_‐Rho. Oxygen atoms=red spheres, T atoms (Si or Al)=light blue spheres and Cu cations=dark blue spheres.

To establish the presence of Cu^2+^ cations in Cu,H‐Rho samples, EPR spectroscopy was conducted on Cu_0.9_H_8.0_‐Rho, Cu_2.1_H_5.6_‐Rho and Cu_4.9_‐Rho samples (see Figure S2 in Supporting Information and Figure 2). The EPR spectrum for Cu_0.9_H_8.0_‐Rho sample showed the best resolution, due to less spin‐spin interactions of neighbouring Cu^2+^ cations in similar environments, therefore it was investigated in detail. The spectrum (Figure 2) showed well resolved hyperfine coupling to ^63/65^Cu giving four peaks at ca. 3000 G in the parallel region of the spectrum (A_||_ hyperfine splitting on g_||_) and a small peak with a deep trough at ca. 3300 G (g_⊥_) with no resolved hyperfine splitting. This was attributed to a planar coordination (g_||_=2.37 and A_||_=153×10^−4^ cm^−1^) of Cu^2+^ cations to three oxygen atoms in the S6R sites as also observed by Rietveld refinement, at a distance of 2.434(15) Å (Figure 3a and see Table S2 in Supporting Information). A previous EPR study on Cu‐Rho by Anderson and Kevan also suggested that copper strongly favours sites in the α‐cage rather than in the octagonal prism.^[33]^ There was an additional broad peak visible at ca. 3150 G and this may indicate the presence of copper cations nearby.

Additionally, XPS analysis was conducted on Cu_4.9_‐Rho before and after heating to determine the oxidation state of copper (Figure S3 in Supporting Information). The XPS spectra for both samples showed a major peak at 934.9 eV identified as Cu^2+^ species. This is in agreement with previously reported XPS Cu 2p_3/2_ binding energy for mazzite (MAZ) zeolite, where Cu^2+^ species were found in *s*6*r* sites.^[34]^ From the deconvolution an additional (unresolved) peak at 932.2 eV might be fitted and assigned to Cu^0^/Cu^+1^ species.^[34]^ On this basis, while the presence of some Cu^+^ cannot be ruled out, at least 70 % of the copper close to the surface of the heated sample was 2+.

Remarkably, upon dehydration at 543 K the samples did not experience the contraction of their unit cells that is observed for zeolite Rho exchanged with other metal cations (e. g. univalent Li, Na, K, Cs or divalent Sr, Ca, Cd)[^9^, ^23^, ^35^, ^36^, ^37^, ^38^] (see Figure S4 in Supporting Information). Structural refinements revealed that the frameworks of Cu,H‐Rho retained a large unit cell parameter, between 14.9947(1) Å and 14.9258(9) Å, although they were better refined in the acentric space group $I\bar{4}3m$ (Table 1 and see Table S1 and Figure S5 in Supporting Information).

**Table 1:** Space group, unit cell parameter and cation site occupancies in dehydrated zeolite Rho as determined by Rietveld refinement.

Sample	Unit cell parameter [Å]	Space group	S6R site		S8R site	
			Frac	Atoms per unit cell	Frac	Atoms per unit cell
H_9.8_‐Rho^[a]^	15.0352(2)	$Im\bar{3}m$	–	–	–	–
Cu_0.9_H_8.0_‐Rho	14.9947(1)	$I\bar{4}3m$	0.1161(17)	0.92(8)	–	–
Cu_2.1_H_5.6_‐Rho	14.9743(5)	$I\bar{4}3m$	0.2635(19)	2.11(1)	–	–
Cu_3.0_H_3.8_‐Rho	14.9352(1)	$I\bar{4}3m$	0.3171(19)	2.53(7)	0.0108(5)	0.51(8)
Cu_4.9_‐Rho	14.9258(9)	$I\bar{4}3m$	0.3592(12)	2.87(4)	0.0423(4)	2.03(1)
Na_9.8_‐Rho^[a]^	14.3771(2)	$I\bar{4}3m$	0.372(11)	2.98(9)	0.539(7)	6.47(8)
Cu_1.0_Na_7.8_‐Rho	14.3449(6)	$I\bar{4}3m$	0.1262(16) (Cu^2+^) 0.21077 (Na^+^)	1.01(7) 1.68	0.2474(15) (Na^+^)	5.94(6)
Cu_3.4_Na_3.0_‐Rho						
acentric phase: Cu_3.0_Na_3.9_‐Rho	14.4052(9)	$I\bar{4}3m$	0.373(13) (Cu^2+^)	2.98(5)	0.166(13) (Na^+^)	3.98(7)
centric phase: Cu_4.9_‐Rho	15.0324(2)	$Im\bar{3}m$	0.289(19) (Cu^2+^)	4.62(7)	–	–

[a] The experimental data is taken from Ref. [22].

In Cu_0.9_H_8.0_‐ and Cu_2.1_H_5.6_‐Rho, the Cu cations occupied only S6R sites and while the unit cell is acentric, *a* remains close to that of the H‐form. In Cu_3.0_H_3.8_‐ and Cu_4.9_‐Rho additional scattering attributed to copper cations was found in the S8R sites (Table 1 and see Table S2, Figure S6 in Supporting Information) but again the effects on the unit cell are minor. Therefore in dehydrated zeolite Rho the Cu cations preferentially occupy S6R sites and even in fully exchanged Cu_4.9_‐Rho there is no strong distortion of the Rho framework.[^36^, ^37^, ^38^]

**Figure 4:**
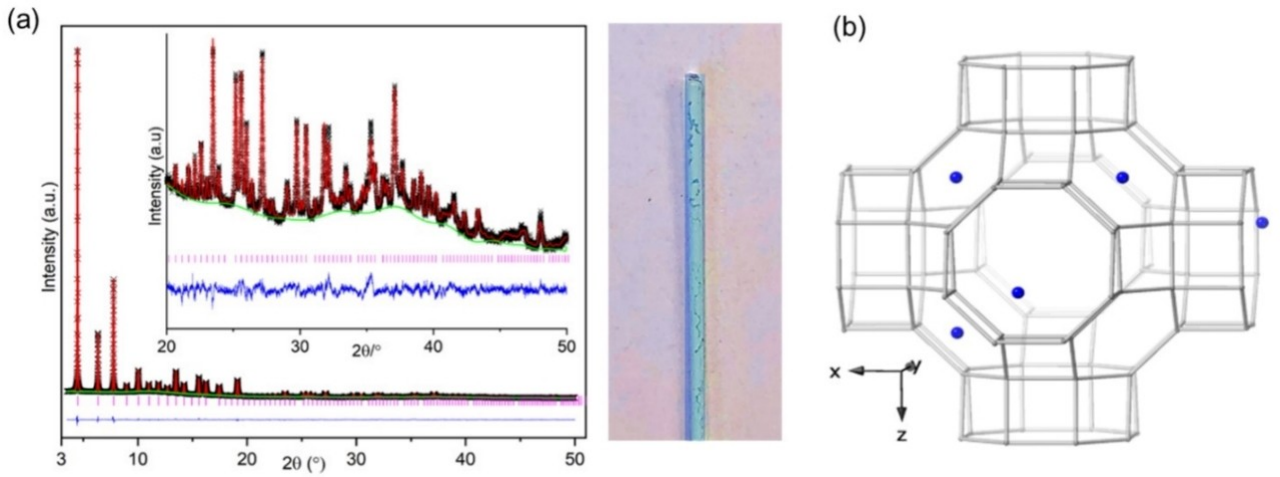
(a) (left) Rietveld plot of synchrotron PXRD data (λ=0.8263980 Å, T=298 K) of dehydrated Cu_4.9_‐Rho (Observed: black, calculated: red, difference: blue, phase: pink and background: green) and (right) a capillary with Cu_4.9_‐Rho sample which remained blue upon dehydration. (b) Generalised model of the structure of zeolite Cu_4.9_‐Rho obtained from synchrotron data. The Cu^2+^ cations=blue spheres. Framework O atoms are omitted for clarity and T−T linkages are represented by grey rods.

Refinement of Cu_4.9_‐Rho (Figure 4) showed that 2.9 of the Cu cations per unit cell occupy a central position in the plane of the *6r* windows where they are coordinated by 3 framework oxygen atoms at a distance of 2.323(4) Å (Figure 3b and see Table S2 in Supporting Information), a similar distance to that previously observed for Cd^2+^ cations in S6R sites in zeolite Rho (2.49(2) Å).^[37]^ Two copper cations per unit cell are present in the S8R sites, situated off‐centre in the ring, coordinated to two O atoms at 2.282(12) Å (see Table S2 in Supporting Information). This occupancy of window sites leaves 4 out of 6 per unit cell unoccupied, which is above the percolation limit. As a result, N_2_ adsorption at 77 K showed high uptake, reaching ca. 9 mmol g^−1^ at p/p_0_=0.9, characteristic of filling the open Rho structure. By contrast, N_2_ adsorption at 77 K on Na_9.8_‐Rho shows no uptake, due to the Na cations blocking the *d8r* windows, rendering the pore volume inaccessible (see Figure S7 in Supporting Information). This combination of undistorted *d8r* windows and relatively low cation occupancy in these windows explains the low O_2_/Ar selectivity and rapid O_2_ diffusion observed previously for Cu_4.9_‐Rho.^[4]^

The structural chemistry of copper in zeolite Rho was studied further in a series of Cu,Na‐Rho samples, prepared as described in the Experimental section and Supporting Information. In contrast to Cu cations, Na cations favourably occupy S8R sites in dehydrated Rho and the electrostatic attraction of Na^+^ cations to framework O atoms of the S8R sites leads to a strong distortion of the structure upon dehydration.[^9^, ^22^] Consequently, it was of interest to examine how a combination of copper and sodium cations would affect the framework of zeolite Rho, and two mixed cation compositions were prepared (Cu_1.0_Na_7.8_‐Rho and Cu_3.4_Na_3.0_‐Rho) (Table 1).

In hydrated form, both samples contain a single phase (see Figure S8 in Supporting Information). Upon dehydration Cu_1.0_Na_7.8_‐Rho adopts $I\bar{4}3m$ symmetry with a unit cell of 14.345(1) Å, similar to fully exchanged zeolite Na_9.8_‐Rho,^[22]^ and the same occupancy of S8R sites, ca. 66 % (Table 1 and see Table S2, Figure S9 and Figure S10 in Supporting Information). The *d8r* windows are very narrow since most of the Na cations occupy preferential S8R sites and this prevents the unit cell from expansion. The Cu cations occupy a slightly off‐centre position in the 6Rs where they are coordinated by three framework O atoms at a distance of 2.132(1) Å (Figure 3c and see Table S2 in Supporting Information).

**Figure 5:**
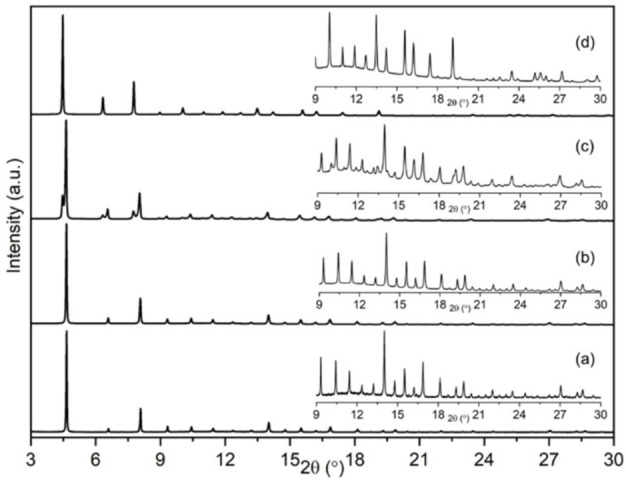
Synchrotron XRD patterns of dehydrated (a) Na_9.8_‐Rho, (b) Cu_1.0_Na_7.8_‐Rho, (c) Cu_3.4_Na_3.0_‐Rho and (d) Cu_4.9_‐Rho with magnified views of 2θ range from 9° to 30°.

Increasing the copper content to Cu_3.4_Na_3.0_‐Rho has a more marked effect, as two different zeolite Rho forms are seen to co‐exist in the dehydrated sample, with different cubic unit cell sizes (Figure 5). To eliminate the possibility of partial dehydration, the sample was kept under vacuum and heated at 623 K for 20 h, double the usual dehydration time, with the same result (see Figure S11 in Supporting Information).

**Figure 6:**
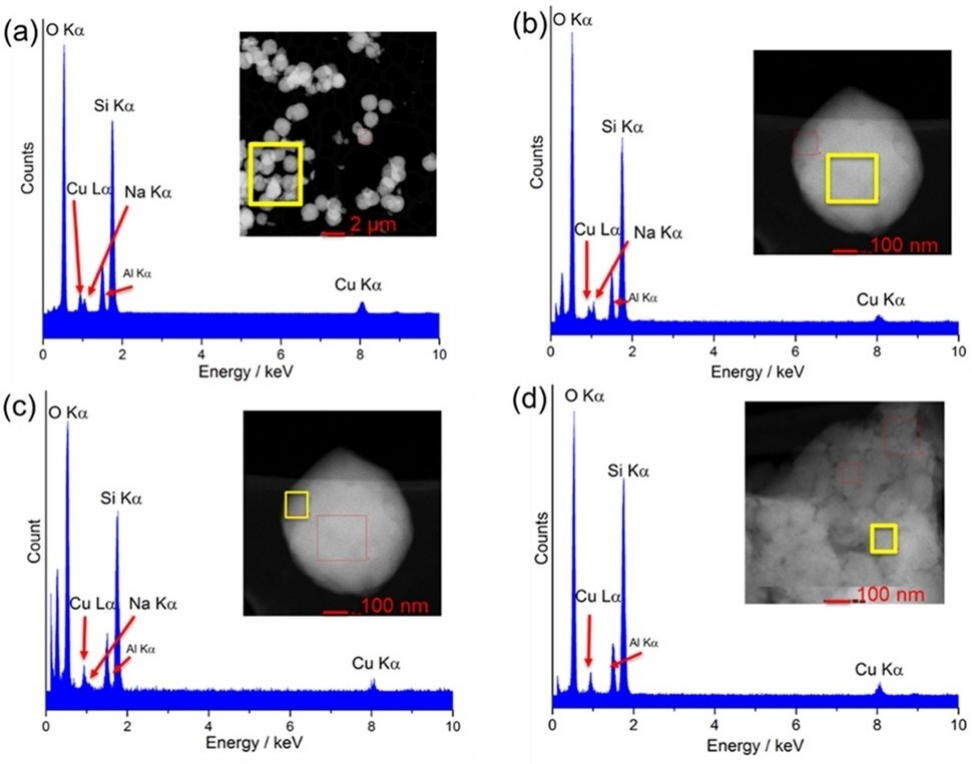
TEM/EDS analysis of the Cu_3.4_Na_3.0_‐Rho crystals. Analysed regions are in yellow squares.

A sample of the heated Cu_3.4_Na_3_‐Rho was investigated by TEM and associated selected area EDS analysis which revealed an uneven distribution of copper and sodium cations (Figure 6). Mixed copper and sodium regions were observed with a range of Cu/Na ratios (Figure 6a and b): in these mixed cation regions the Cu cations tend to concentrate close to the edge of the crystals (Figure 6c). Additionally, copper‐only regions were observed (Figure 6d). This indicates that Cu cations migrate over hundreds of nanometres, and is consistent with the powder diffraction data that indicates crystalline domains of at least these dimensions are present in the exsolved mixture of phases.

**Figure 7:**
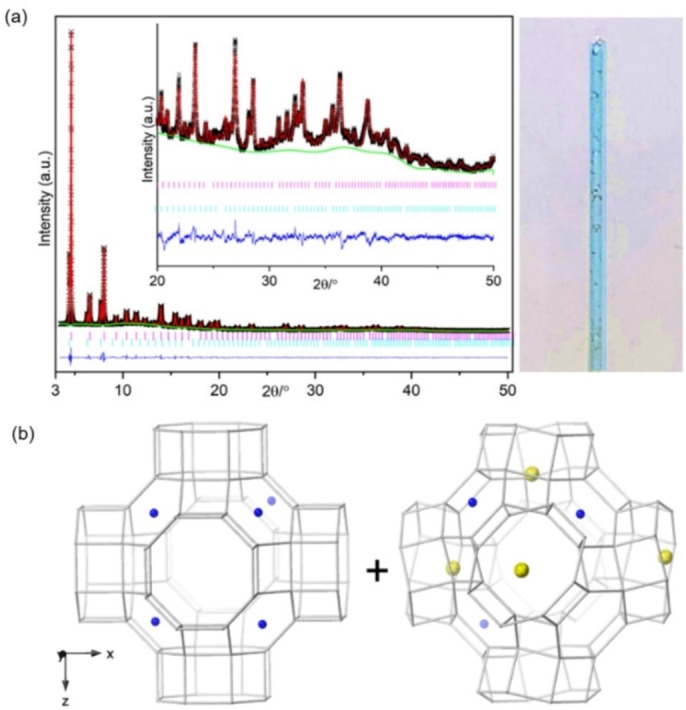
(a) (left) Rietveld plot of synchrotron PXRD data (λ=0.8263980 Å, T=298 K) of dehydrated Cu_3.4_Na_3.0_‐Rho (Observed: black, calculated: red, difference: blue, phase: pink ($I\bar{4}3m$ symmetry) and bright blue ($Im\bar{3}m$ symmetry), background: green) and (right) a capillary with the sample which remained blue upon dehydration. (b) Generalised model of the two coexisting structures (centric on the left and acentric on the right) of zeolite Cu_3.4_Na_3.0_‐Rho obtained from synchrotron data. The Cu^2+^ cations=blue spheres, the Na^+^ cations=yellow spheres. Framework O atoms are omitted for clarity and T−T linkages are represented by grey rods.

Two‐phase Rietveld refinement of synchrotron data identified that this Cu_3.4_Na_3.0_‐Rho consists of an acentric phase ($I\bar{4}3m$ symmetry, Cu_3.0_Na_3.9_‐Rho, *a*=14.4052(9) Å) and a centric phase ($Im\bar{3}m\;$ symmetry, Cu_4.9_‐Rho, *a*=15.0324(2) Å) (Table 1 and Figure 7). The centric phase accounts for 20 % of the overall composition ($I\bar{4}3m$ :$Im\bar{3}m$ =4 : 1) and the average unit cell composition was estimated as Cu_3.4_Na_3.0_‐Rho. The acentric phase was found to contain ca. 4 Na cations in S8R sites, and 3 Cu cations in the S6R sites (Table 1). The structure of the centric phase exhibits the unit cell size of fully open zeolite Rho. The *8r* windows are too large for extra‐framework cations to coordinate with framework oxygen atoms, hence the 4.6 Cu cations were found in S6R sites. Nevertheless, since the phase fraction of acentric phase is 4× higher than centric phase it is very likely that some Cu cations are present in the acentric phase.

This PXRD and TEM/EDS analysis suggests that an exsolution process has occurred during dehydration. Exsolution commonly occurs upon cooling of solid solutions[^39^, ^40^] but is rarely seen in zeolites. In zeolitic materials that have been reported, such as those of zeolite P, amicite and merlinoite, it is, as in this case, observed upon dehydration of a zeolite with a flexible framework.[^25^, ^41^, ^42^] During dehydration, cations must be able to diffuse through the structure on a micron length scale to achieve thermodynamically favourable locations. Since phase separation is associated with an entropy loss, this process must result in a reduction in enthalpy, as the zeolite distorts to coordinate cations more closely.

Upon dehydration of Cu_3.4_Na_3.0_‐Rho, the enthalpy can be reduced by concentrating Na cations within a distorted Rho where the Na cations can occupy narrow *d8r* windows and achieve better coordination. This is achieved by intrazeolitic cation exchange for copper cations. The copper cations that leave form Cu_4.9_‐Rho, in which they can be well coordinated in S6R sites without the framework needing to distort. To investigate the strength of the tendency of Na cations in copper‐rich samples to drive exsolution, an additional sample, Cu_4.3_Na_1.0_‐Rho, with a sodium content of 1 cation per unit cell was prepared. This also showed exsolution upon dehydration (see Figure S12 in Supporting Information). Furthermore, previously reported Na_4.5_H_5.3_‐Rho sample (a=14.3447(2) Å, see Figure S13 in Supporting Information)^[22]^ did not show exsolution upon dehydration indicating a requirement of presence of Cu cations in zeolite Na‐Rho for the exsolution to occur. The exsolution occurs in Rho because of the flexibility of its framework, and its ability to adopt two very different conformations that are well adapted to the different cation types. Exsolution phenomena during dehydration are likely to be common within flexible zeolite structures for mixed cation zeolites of certain compositional ranges where the cations have very different ionic radii, charge or electronegativity.

### CO_2_ adsorption isotherms and kinetics

The synthetic and structural investigation showed that it is possible to control the size of the *d8r* windows in Rho and their fractional cation occupancy through exchange of Cu cations into zeolite Na_9.8_‐Rho. The small window size in Na_9.8_‐Rho results in very high CO_2_/CH_4_ selectivity but prohibitively slow adsorption kinetics. By relocating cations away from the windows, and thereby enlarging their free diameter, we expect an improvement of the kinetics while retaining good selectivity. The CO_2_ adsorption isotherms, the kinetics of CO_2_ adsorption and the CO_2_/CH_4_ selectivity under dynamic conditions were therefore measured for selected Cu,Na‐Rho materials and compared with the Na_9.8_‐ and Cu_4.9_‐Rho end‐members.

**Figure 8:**
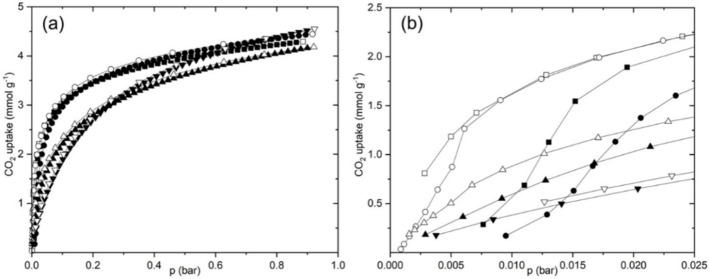
CO_2_ isotherms at 298 K on Na_9.8_‐Rho (▪), Cu_1.0_Na_7.8_‐Rho (•), Cu_3.4_Na_3.0_‐Rho (▴) and Cu_4.9_‐Rho (▾). Adsorption, closed symbols; desorption, open symbols.

CO_2_ adsorption isotherms (with desorption branches) were measured at 298 K on Na_9.8_‐Rho, Cu_1.0_Na_7.8_‐Rho, Cu_3.4_Na_3.0_‐Rho and Cu_4.9_‐Rho (Figure 8). As described previously, the sodium end‐member shows high uptakes of 3.1 mmol g^−1^ at 0.1 bar and 4.2 mmol g^−1^ at 0.9 bar as a result of the strong interaction of CO_2_ with the abundant Na cations. At very low pressures (<0.025 bar) the uptake increases sigmoidally during adsorption, characteristic of structural changes (the zeolite expands) which result in hysteresis on the desorption branch. Notably, the equilibration time for Na‐Rho is long, especially at low *p*_CO2_, which is explained by the cation gating effect of Na cations, requiring cation movement to allow uptake.^[22]^

By contrast, the uptake on Cu_4.9_‐Rho is rapid, Type I and fully reversible. It achieves a lower uptake at 0.1 bar than Na_9.8_‐Rho (1.8 mmol g^−1^), but similar uptake at 0.9 bar (4.5 mmol g^−1^). Indeed, the isotherm characteristics are similar to those of H_9.8_‐Rho,^5^ which also possesses an open structure in the dehydrated form. The low uptake at lower pressures indicates weak electrostatic interactions of CO_2_ with the copper cations.

Introduction of one Cu^2+^ cation into Na_9.8_‐Rho has little effect on the CO_2_ adsorption properties so that it also shows hysteresis at low pressures caused by the cation gating effect. In this material there remains around one Na cation per *d*8*r* window,^[22]^ so that cation migration is still required to permit uptake. By contrast, uptake is much faster for the adsorption of CO_2_ on the two‐phase Cu_3.4_Na_3.0_‐Rho sample and negligible hysteresis was observed. This two‐phase material, as a result from exsolution upon dehydration, exhibits Type I adsorption which indicates that both phases display this type of adsorption behaviour. Type I behaviour is expected for the minority Cu‐rich phase, as it is close to the Cu end‐member. For the Na‐rich phase, which has a unit cell size similar to that of the Na end‐member (*a*=14.4052(9) Å cf. 14.3771(2) Å), there are fewer cations in window sites than in the Na end‐member (4/6 windows occupied), which introduces sufficient permeation to strongly reduce the effect of cation gating and thus yield Type I adsorption behaviour. (This is also implied by the N_2_ adsorption at 77 K, which at 3.2 mmol g^−1^ (at 0.1 bar) is more than that expected for the minority Cu‐rich phase alone, see Figure S14 in Supporting Information.) At 0.1 bar the uptake of CO_2_ of the mixed phase is higher than for Cu_4.9_‐Rho, but well below that of the Na_9.8_‐Rho: at 1 bar the CO_2_ uptake is close to that of the Na_9.8_‐Rho, ca. 4.2 mmol g^−1^.

Isosteric heats of adsorption were determined for Cu_3.4_Na_3.0_‐Rho and Cu_4.9_‐Rho and compared with those reported previously for Na_9.8_‐Rho,^[9]^ (see Figures S15 and S16 in Supporting Information). Na_9.8_‐Rho has a heat of adsorption of 38–42 kJ mol^−1^ over range of 1.5‐3.5 mmol g^−1^. The Cu_3.4_Na_3.0_‐Rho and Cu_4.9_‐Rho materials showed lower heats of adsorption, 30–38 kJ mol^−1^ over the same range of uptakes, which is attributed primarily to the presence of fewer cations.

The kinetics and CO_2_/CH_4_ selectivity were measured by a combination of ZLC and extended ZLC measurements. The ZLC measurements of the very slow uptake of CO_2_ on Na‐Rho have been reported previously but were repeated for consistency.^[22]^ At 308 K, with a 4.0 mg sample and a 3 mL min^−1^ flow of 10 % CO_2_ in He, the equilibrium time is in excess of 1 h, and desorption from a sample loaded with 1.86 mmol g^−1^ is strongly kinetically limited and cannot be fitted in a straightforward way, because the structure undergoes structural changes. At low loadings, the last molecules to be desorbed show a D/R^2^ value of 5.2×10^−5^ s^−1^ (see Figures S17 and S18 in Supporting Information and Table 2).

**Table 2:** Comparison of uptakes of CO_2_ and CH_4_, selectivity and kinetic diffusion parameters for Na_9.8_‐Rho, Cu_4.9_‐Rho and Cu_3.4_Na_3.0_‐Rho in flowing 10 % CO_2_/40 % CH_4_/He at 308 K.

Sample	Uptake of CH_4_ [mmol g^−1^]	Uptake of CO_2_ [mmol g^−1^]	α (CO_2_/CH_4_)	D/R^2^ [s^−1^]
Na_9.8_‐Rho	0.05	2.55	209	5.2×10^−5^
Cu_4.9_‐Rho	0.17	0.85	20.1	2.3×10^−2^
Cu_3.4_Na_3.0_‐Rho	0.15	1.27	33.7	2.5×10^−4^

**Figure 9:**
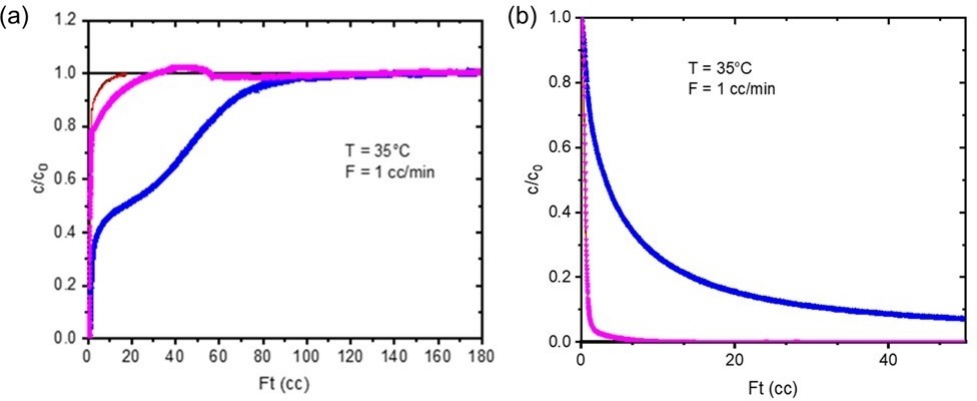
Binary breakthrough experiment for Na_9.8_‐Rho in 10 % CO_2_/40 % CH_4_/He at 308 K. Blue symbols: CO_2_, pink symbols: CH_4_, black/red line: CO_2_/CH_4_ blank experiment. (a) Fast breakthrough of CO_2_ during adsorption indicates poor kinetics. (b) No CH_4_ is observed on desorption (overlap with blank experiment), indicating good selectivity for CO_2_.

Breakthrough curves were measured for 36.8 mg Na_9.8_‐Rho, in 40 % CH_4_ in He (2 mL min^−1^) and in a mixture of 10 % CO_2_/40 % CH_4_ in He (1 mL min^−1^), typical of some CO_2_‐rich natural gases and biogas (Figure 9). No CH_4_ is adsorbed in the first experiment. For the mixture, the poor CO_2_ adsorption kinetics result in rapid breakthrough of this component, so that under these conditions there is no period where pure CH_4_ is produced. Some small amounts of CH_4_ are taken up in the mixed gas experiment, because the structure opens up as CO_2_ is adsorbed. After saturation, desorption reveals a high CO_2_ uptake, as expected (2.55 mmol g^−1^) and very high selectivity (209), as evidenced by the very small amount of CH_4_ in the desorption curve.

**Figure 10:**
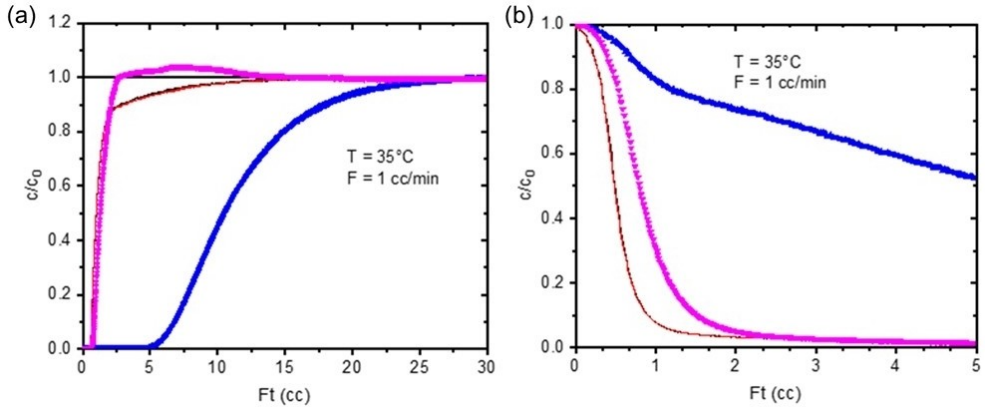
Binary breakthrough experiment for Cu_4.9_‐Rho in 10 % CO_2_/40 % CH_4_/He at 308 K. Blue symbols: CO_2_, pink symbols: CH_4_, black/red line: CO_2_/CH_4_ blank experiment.

The adsorption and desorption of CO_2_ on the Cu_4.9_‐Rho sample is, by comparison with Na_9.8_‐Rho, very fast. The rapid kinetics of Cu_4.9_‐Rho can be explained by its nearly circular windows, and the low concentration of blocking cations near them. ZLC measurements of desorption at 308 K from an equilibrated 10 % CO_2_/He‐loaded sample indicate desorption is nearly complete after 1–2 minutes, and the entire desorption process can be described using the standard ZLC model for linear isotherms with a D/R^2^ of 2.3×10^−2^ s^−1^, some three orders of magnitude higher than observed for Na‐Rho (see Figures S19 and S20 in Supporting Information and Table 2). The breakthrough curve of methane at 308 K (40 % CH_4_ in He) shows some uptake (0.20 mmol g^−1^), which is possible due to the circular and unblocked *d8r* windows (Figure 10). The rapid CO_2_ diffusion enables the production of pure CH_4_ in a mixed gas breakthrough experiment, but with lower CO_2_ capacity than Na_9.8_‐Rho because of the weaker interaction, showing an uptake of 0.85 mmol g^−1^ and a selectivity of 20. In the desorption profile a clear difference between the blank experiment can be seen for both components, showing that some CH_4_ is desorbing, which is indicative of a reduced selectivity compared to Na‐Rho.

**Figure 11:**
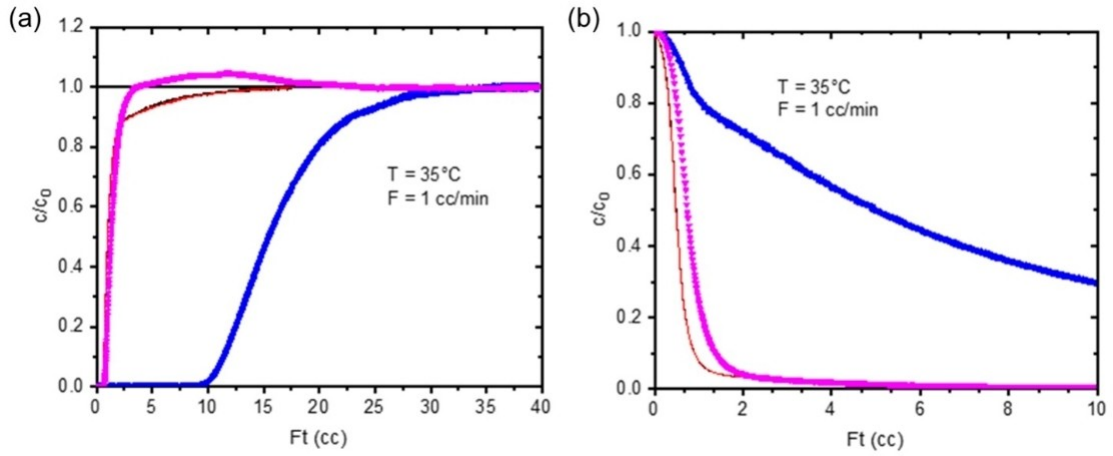
Binary breakthrough experiment for Cu_3.4_Na_3.0_‐Rho‐Rho in 10 % CO_2_/40 % CH_4_/He at 308 K. Blue symbols: CO_2_, pink symbols: CH_4_, black/red line: CO_2_/CH_4_ blank experiment.

For the mixed Cu_3.4_Na_3.0_‐Rho sample, ZLC measurements at 308 K show rapid uptake at 1 % CO_2_, and the desorption is only kinetically limited at very low loadings with a D/R^2^ of 2.5×10^−4^ s^−1^ (see Figures S21 and S22 in Supporting Information and Table 2). This accounts for the lack of hysteresis in the adsorption isotherms and is due to the open Cu‐Rho phase and the ability for gases to permeate through the Cu,Na‐Rho phase. Breakthrough curves for methane show some CH_4_ can adsorb (0.21 mmol g^−1^), and the mixed gas breakthrough curve (Figure 11) shows an extended period where pure methane is produced compared to Cu‐Rho, due to the greater capacity of Cu_3.4_Na_3.0_‐Rho than Cu_4.9_‐Rho. The zeolite shows an adsorption capacity of 1.39 mmol g^−1^ and a selectivity of 33.7, a significant improvement over Cu_4.9_‐Rho. Given that some 20 % of the exsolved mixture is essentially Cu‐Rho, this indicates that the Cu_3.4_Na_3.0_‐Rho itself would possess a selectivity significantly above 35.

The results show that the kinetics and selectivity of CO_2_ adsorption by zeolite Rho are strongly dependent on the extra‐framework composition. There is a trade‐off between the uptake rate, which is faster when fewer windows are blocked by cations that occupy S8R sites, and selectivity, which is enhanced when there are more cations in S8R sites and the windows are more elliptical and therefore narrower, giving shape selectivity for CO_2_ against the larger CH_4_. It should be noted that this will be modified at different CO_2_ concentrations, because the presence of CO_2_ is known to cause structural changes in zeolite Rho.

## Conclusion

A series of Cu,H‐Rho samples have been prepared, up to the fully exchanged Cu_4.9_‐Rho, by Cu^2+^ ion exchange of the ammonium‐form, followed by deammoniation. In the dehydrated form, the Cu,H‐Rho samples only show a slight decrease in unit cell size, remaining close to the fully open framework ‘15 Å’ form. Cu cations show a strong preference for the S6R sites, where they are located in trigonal coordination in the plane of the 6Rs, up to ca. 3 per unit cell, although some are also found in the S8R sites in samples with higher Cu content. The preferred occupancy of the S6R sites by Cu cations stabilises the open framework, which is unusual for dehydrated cationic forms other than H_9.8_‐Rho.

Cu,Na‐Rho samples were also prepared by ion exchange of Na_9.8_‐Rho, with the aim of improving the kinetics of CO_2_ adsorption of Na‐Rho and the CO_2_/CH_4_ separation of Cu‐Rho. Inclusion of one Cu cation per unit cell (Cu_1.0_Na_7.8_‐Rho) does not change the structural behaviour upon dehydration and like the Na_9.8_‐Rho gives a strongly distorted unit cell with full occupancy of *d8r* windows with Na cations. However, inclusion of 3.4 Cu cations per unit cell (Cu_3.4_Na_3.0_‐Rho) gives a solid that upon dehydration exhibits exsolution to give Cu_4.9_‐Rho and Cu_3.0_Na_3.9_‐Rho. This is observed by PXRD and by selected area EDS in TEM, which suggests long‐range diffusion of Cu and Na cations (of the order of 0.5 μm) and the generation of Cu‐rich Rho near the surface of particles. This exsolution behaviour is made possible by the flexibility of the structure, and its resulting ability to adapt to give sites of appropriate coordination geometry for cations depending on their size, charge and electronic structure. While the smaller cell (14.4052(9) Å) gives distorted S8R sites favourable for Na cations, the open (15.0324(2) Å) cell is favoured by Cu cations in the S6R sites.

The CO_2_ adsorption properties of the Cu‐, Na‐ and Cu,Na‐Rho materials are strongly dependent on composition, and there are three orders of magnitude variation in the measured diffusion time constants: the very slow adsorption of Na_9.8_‐Rho results from Na cations blocking elliptical windows, while the fast adsorption of Cu_4.9_‐Rho is possible due to its circular unblocked windows. Although Na_9.8_‐Rho shows very high selectivity for CO_2_/CH_4_ adsorption in dynamic breakthrough experiments, its slow kinetics for CO_2_ adsorption do not allow for clear separation of the two gas components. The open structure of Cu_4.9_‐Rho, on the other hand, allows very fast CO_2_ uptake and the production of pure CH_4_, but with reduced capacity at 0.1 bar CO_2_ and lower selectivity. The exsolved Cu_3.4_Na_3.0_‐Rho sample shows fast diffusion, in the Cu,Na‐Rho as well as the Cu‐Rho phases, because Na cations no longer block all the windows in the mixed cation form. Furthermore, it shows enhanced uptake of CO_2_ and produces more pure CH_4_ in the breakthrough tests, as well as being more selective for CO_2_ over CH_4_ overall.

These results confirm that the performance of mixed cation zeolite Rho in selective gas adsorption is highly sensitive to the composition, charge, size and electronic structure of its extra‐framework cations, and this is at least partly because of the flexibility of its framework. This offers many opportunities for the design of task‐specific Rho‐based adsorbents.

## Experimental Section

Zeolite Na,Cs‐Rho (RHO; (Na,Cs)_9.8_Al_9.8_Si_38.2_O_96_) was synthesised in the presence of the crown ether, 18‐crown‐6, using a previously reported procedure (see S1 in the Supporting Information).^[43]^ The organic was removed by calcination at 823 K in flowing oxygen gas. The synthesised zeolite Na,Cs‐Rho was fully exchanged to the ammonium form with 3 M ammonium chloride solution at 333 K, eight times for 5 h. Subsequently the ammonium form was converted to sodium form by extended cation exchange treatments at 353 K using 10 wt % metal nitrate solutions. To prepare mixed cation Cu,NH_4_‐Rho and Cu,Na‐Rho samples the ion exchange with low concentration (0.05 M) copper nitrate solution at 333 K for 2–4 h was performed until desired compositions were achieved. A low concentration of copper nitrate solution was required to avoid precipitation of copper hydroxide on the zeolite surface.^[44]^ The mixed Cu,H‐Rho samples were prepared via deammoniation of the Cu,NH_4_‐samples by heating under shallow bed conditions in dry flowing nitrogen at 823 K for 12 h. The compositions of mixed cation samples, determined by Rietveld refinement of diffraction data, are given in the forms Cu_x_H_y_‐Rho or Cu_x_Na_y_‐Rho, where *x* and *y* are the numbers of Cu^2+^ and H^+^ or Na^+^ cations per unit cell, respectively.

The sample of Cu_3.4_Na_3.0_‐Rho for TEM/EDS analysis was crushed in a mortar and pestle, dispersed in ethanol and deposited on a holey carbon film supported on a copper grid. EDS measurements were carried out using a spherical aberration corrected (Cs‐corrected) FEI Titan Themis 200 transmission electron microscope equipped with a high brightness Schottky X‐FEG emitter and operated at 200 kV with a convergence angle of 20 mrad.

For EPR analysis, Cu_1.0_H_7.8_‐Rho, Cu_2.1_H_5.6_‐Rho and Cu_4.9_‐Rho samples were packed into 25 cm long, 0.4 cm diameter quartz EPR tubes (1 cm length of sample) and dehydrated on the glass line at 623 K at 5×10^−5^ mbar for 10 h before flame‐sealing. Measurement was performed in an ELEXSYS Super High Sensitivity Probehead (Bruker ER4122SHQE) using a Bruker EMX 10/12 spectrometer operating at 9 GHz with 100 kHz modulation frequency. The EPR spectra were recorded at 295 K using 2 mW microwave power, a 3000 G field sweep centred at 2700 G with 3000 points resolution, a time constant and conversion time of 40.96 ms each, and a modulation amplitude of 3 G.

For XPS analysis, Cu_4.9_‐Rho was measured before and after heating at 453 K under vacuum for 10 h, using a Scienta 300 spectrometer operating at or below 1×10^−9^ mbar. The X‐ray source is an SPECS monochromated Al K_α_ source (photon energy 1486.6 eV) operating at approx. 12 kV and 200 watts. The instrument maintains a pass energy set to 150 eV for all spectra. Survey scans were collected at a dwell time of 133 msec, step size 200 meV and 2 scans were added. Detailed scans were 2 to 5 scans depending on the S : N ratio, a dwell time of 533 msec and a step size of 20 meV. Commercially available CuO was measured as a standard. The FWHM of the Ag 3d^5^ peak at 368.4 eV is routinely below 0.55 eV with a similar value for Au 4f^7^ at 84 eV and experimental drift as a function of time is negligible over a period of 24 h.

The crystallinity of as‐prepared, cation‐exchanged and dehydrated samples was confirmed by laboratory powder X‐ray diffraction (PXRD) using a Stoe STAD I/P diffractometer with Cu K_α1_ X‐radiation (1.54056 Å). To determine the structure of dehydrated zeolites, the powders were loaded into 0.7 mm quartz capillaries and dehydrated at 623 K at 5×10^−5^ mbar on a glass vacuum line for 10 h. The PXRD patterns of the dehydrated samples were obtained from these loaded and sealed capillaries. Additionally, synchrotron X‐ray powder diffraction at beamline I11 of the Diamond Light Source was performed on Cu,Na‐Rho and Cu_4.9_‐Rho samples.

The structures were determined by Rietveld refinement against the PXRD, using the GSAS suite of programs.^[45]^ For the zeolite Rho, $Im\bar{3}m\;$ and $I\bar{4}3m\;s$ ymmetries, starting framework models were adapted from the literature with the unit cell modified to that derived from the diffraction patterns.^[22]^ Samples with unit cell parameter *a* equal to and above 15.0 Å were refined in $Im\bar{3}m\;$ symmetry and those below 15.0 Å in $I\bar{4}3m\;$ symmetry. The background for all patterns was fitted by an 8‐term shifted Chebyshev function. The framework atomic positions were initially refined with geometric restraints on T−O (T=Si or Al; 1.64±0.02 Å) and O−O (2.65±0.02 Å) distances to maintain regular tetrahedral coordination. Three starting extra framework cation sites: single 6‐ring (S6R), single 8‐ring (S8R) and double 8‐ring (D8R) were estimated from literature models and their fractional occupancies and atomic coordinates refined.^[22]^ No electron density in D8R sites was found for any refined samples. For the Cu,H‐Rho series and Cu_4.9_‐Rho sample, all electron density in S6R and S8R sites was attributed to Cu^2+^ cations. For mixed cation Cu,Na‐Rho samples, the Na^+^ and Cu^2+^ cations can simultaneously occupy S6R and S8R sites, therefore a combination of Rietveld refinement and compositional analysis was applied. For Cu_1.0_Na_7.8_‐Rho and Cu_3.4_Na_3.0_‐Rho samples, the Na^+^ cations are known to preferentially occupy the S8R site[^9^, ^22^] in the dehydrated Rho structure therefore, they were firstly refined in that site and Cu^2+^ cations in the S6R site. Additionally, from the Fourier mapping analysis for Cu_1.0_Na_7.8_‐Rho extra scattering was observed in the S6R site, therefore Na^+^ cations were added at the position at which they occupy this site in Na_9.8_‐Rho and refined. The crystallographic data for all RHO structures is given in the Supporting Information and *cif* files.

CO_2_ and N_2_ adsorption isotherms were measured volumetrically at 298 K and 77 K, respectively, using a Micromeritics ASAP 2020 Gas Adsorption Analyzer connected to a Julabo F25 Chiller Unit. The samples were activated to 573 K at 5 K min^−1^ under vacuum and held at this temperature for 6 h before cooling and measurement. At each adsorption or desorption step the pressure was sampled every 7 s until no further change is observed, so that step times ranged from 10 to 100 min.

Additionally, high pressure CO_2_ adsorption isotherms from 0–10 bar at 283, 298 and 313 K, used to calculate the isosteric heats of adsorption, were measured gravimetrically on a Hiden Intelligent Gravimetric Analyzer (IGA). All samples were activated at 573 K for 6 h prior to measurements. The mass change for each adsorption/desorption step was followed, and a final reading was taken when it had reached 98 % of the asymptotic equilibrium value or after 90 min, whichever was shorter. The isosteric heats of adsorption for Cu_4.9_‐Rho and Cu_3.4_Na_3.0_‐Rho samples were determined using the Clausius‐Clapeyron equation at uptakes from 1.5 to 3.5 mmol g^−1^. The isotherms were first fitted by virial equations using Desmos software^[46]^ and subsequently pressures giving specific uptakes were obtained from these fits. The heats of adsorption for Na_9.8_‐Rho sample were sourced from previously published data.^[9]^

The Zero‐Length Column (ZLC) experimental setup is described in detail in ref..^[47]^ In summary, small amounts (5–10 mg) of (Na,Cu)‐Rho sample were packed into a 1/8” stainless steel union (Swagelok®), fitted with two porous metal discs to keep the powder in place. The column and gas connections are placed either within an oven (Carbolite) with thermostatic control (Eurotherm) or inside a cooling jacket, connected to a thermostatic bath for temperature control (Julabo F‐25). The pure helium carrier and dosing gas mixtures (1–10 vol.% CO_2_ in helium) are supplied through mass flow controllers (Brooks Instrument) and a combination of four solenoid valves is used to direct either of the two gas streams to the ZLC. Both helium (BOC, CP grade, 99.999 % purity) and CO_2_ (BOC, 99.8 % purity) are additionally dried using columns packed with a combination of silica gel and zeolite 5 A molecular sieve. The gas leaving the ZLC is analysed by mass spectrometry (Dycor Residual Gas Analyzer, Ametek Process Instruments). Prior to ZLC measurements, the sample was activated overnight at 473 K under a flow of helium.

The ZLC method is in essence a chromatographic technique, whereby the desorption of a previously equilibrated adsorbent is monitored.[^48^, ^49^] Equilibration occurs in the dilute mixture of adsorbate in inert carrier, whereas desorption takes place in the pure inert carrier. The small amount of sample allows for neglecting external mass and heat transfer resistances and the short length of the column allows for treating the system as a well‐mixed cell (CSTR), due to negligible axial concentration gradients.^[50]^ Pressure drops are additionally assumed to be negligible and the system is treated as being isothermal.

Breakthrough experiments were used to assess the materials’ potential for gas separation. In these experiments, a special “elongated” version of the zero‐length column (E‐ZLC) is used. The E‐ZLC consists of a Swagelok 1/8′′ bulkhead union with an internal diameter of 2.286 mm and a length of 25.9 mm. As a result the columns can hold up to five times the amount of sample that is normally used in a typical ZLC experiment, allowing a clear identification of the separation performances. Apart from the extended column, the experimental apparatus used for this study is identical to the ZLC setup described above. The experiments were carried out at 308 K at ambient pressure and at different flow rates, i. e. 1, and 2 mL min^−1^, in a gas mixture which is taken as representative for a CO_2_ containing natural gas, with composition 10 % CO_2_/40 % CH_4_ (BOC, 99.995 % purity)/50 % He. In order to minimize the pressure drop across the column, the samples were made as binderless pellet fragments of ca. 2 mm in size. The amounts of material used was 41.2 mg for Cu_4.9_‐Rho, 37.6 mg for Cu_3.4_Na_3.0_‐Rho and 36.8 mg for Na_9.8_‐Rho.

The selectivity is described by the following Equation (1), where $q_{i}$ is the equilibrium amount adsorbed for component *i* at its partial pressure,$\;p_{i}$ in the binary system. The equilibrium amounts adsorbed are determined by appropriate integration of the desorption curves of the breakthrough experiments.

(1) $$\alpha_{{CO}_{2}/{CH}_{4}}=\frac{q_{{CO}_{2}}/p_{{CO}_{2}}}{q_{{CH}_{4}}/p_{{CH}_{4}}}\;$$

To enable analysis of both breakthrough and ZLC results, blank runs were also carried out.^[51]^ These consist of repeating the column experiments under the same conditions as described above, but without adsorbent. In this case the column is filled with 2 mm glass beads to give a pressure drop and void fraction close to that observed in the presence of the samples. This allows for measuring the dead volume and intrinsic kinetics of the system when no adsorption occurs.

## Conflict of interest

The authors declare no conflict of interest.

## Supporting information

As a service to our authors and readers, this journal provides supporting information supplied by the authors. Such materials are peer reviewed and may be re‐organized for online delivery, but are not copy‐edited or typeset. Technical support issues arising from supporting information (other than missing files) should be addressed to the authors.

Supporting Information
