# Supplementary material for: Cation Ordering and Exsolution in Copper‐Containing Forms of the Flexible Zeolite Rho (Cu,M‐Rho; M=H, Na) and Their Consequences for CO2 Adsorption
Source: Chemistry. 2021 Aug 6;27(51):13029–39. doi: 10.1002/chem.202101664 (PMC8518693; doi:10.1002/chem.202101664)
Supplement: Supplementary file 1 — Supporting Information [file CHEM-27-13029-s001.pdf]

Chemistry–A European Journal

Supporting Information

Cation Ordering and Exsolution in Copper-Containing Forms of the Flexible Zeolite Rho (Cu,M-Rho; M=H, Na) and Their Consequences for CO_2 Adsorption

Magdalena M. Lozinska,* Sophie Jamieson, Maarten C. Verbraeken, David N. Miller, Bela E. Bode, Claire A. Murray, Stefano Brandani, and Paul A. Wright*

S1. Synthesis of zeolite Na,Cs-Rho

Zeolite Na,Cs-Rho was synthesised from the gel composition: 0.31 NaOH : 0.02 Cs_2O: 1.0 Al_2O_3 : 3.1 SiO_2 : 0.16 (18-crown-6) : 14 H_2O, according to published procedures.[1] The starting mixture was prepared by dissolving 1,4,7,10,13,16-hexaoxacy-clooctadecane (4 mmol; Sigma-Aldrich), cesium hydroxide (5 mmol; Sigma-Aldrich) and sodium hydroxide (8 mmol; Fisher Chemicals) in distilled water. Sodium aluminate (26 mmol; BDH Chemicals Ltd.) followed by colloidal silica, Ludox AS-40 (208 mmol; Sigma-Aldrich) was added and the mixture stirred until homogeneous. The gel formed was aged at room temperature for 24 hours in a closed polypropylene bottle under continuous stirring. The crystallization was carried out under static conditions in the same closed polypropylene bottle for 8 days at 383 K. After reaction, the solid obtained was filtered, washed with distilled water then dried at 373 K overnight.

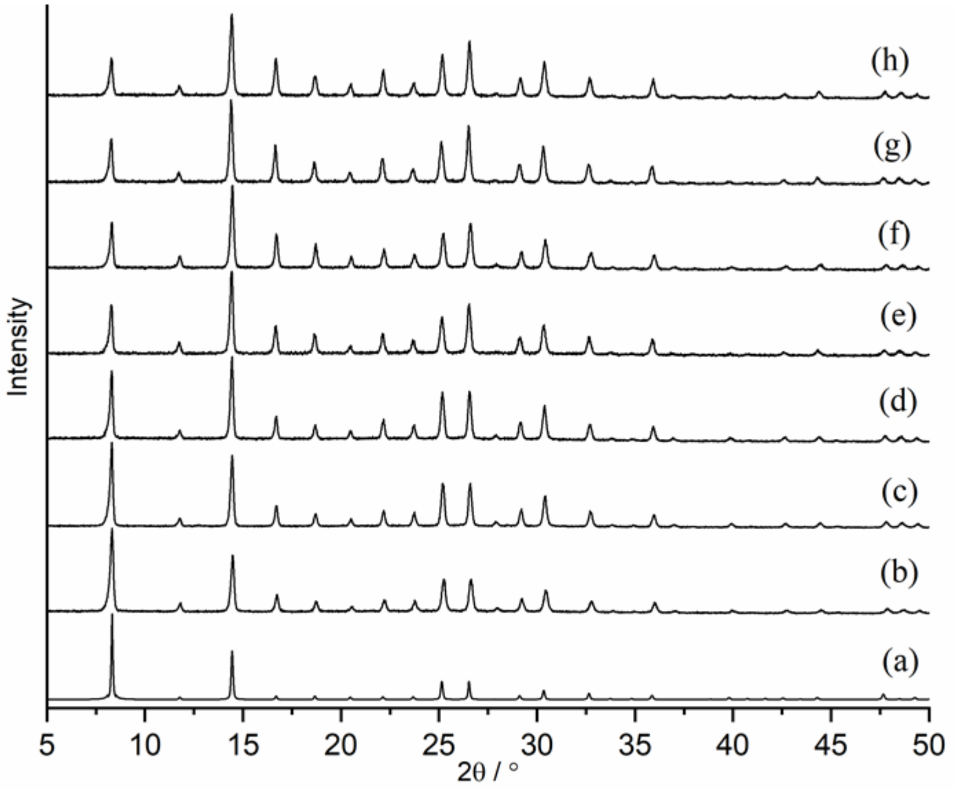

Figure S1. XRD patterns of hydrated (a) simulated $H_{9.8}$-Rho, (b) $Cu_{0.9}(NH_4)_{8.9}$-Rho, (c) $Cu_{0.9}H_{8.9}$-Rho, (d) $Cu_{2.1}(NH_4)_{5.6}$-Rho, (e) $Cu_{2.1}H_{5.6}$-Rho, (f) $Cu_{3.0}(NH_4)_{3.8}$-Rho, (g) $Cu_{3.0}H_{3.8}$-Rho and (h) $Cu_{4.9}$-Rho.

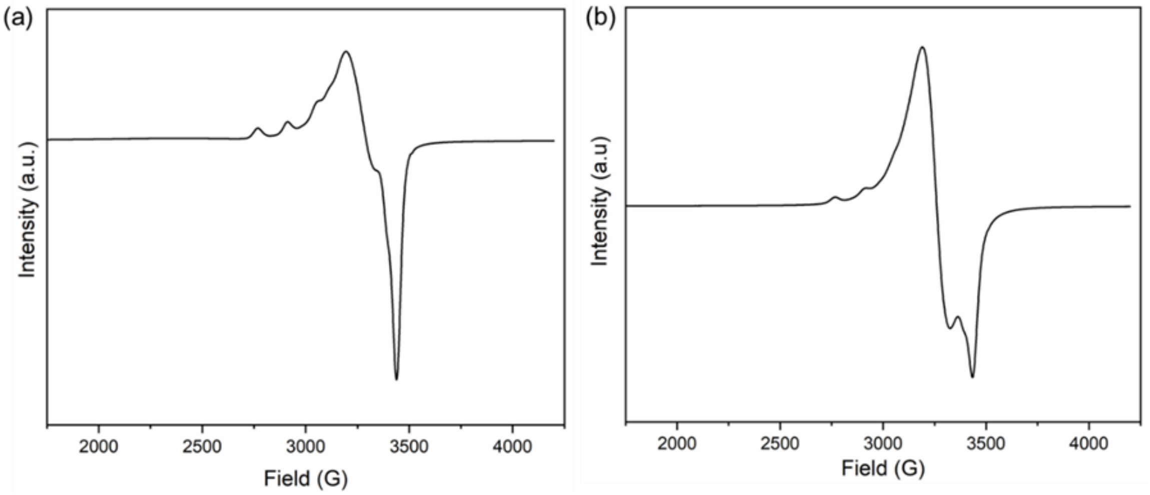

Figure S2. EPR spectra (295 K) of dehydrated (a) $Cu_{2.1}H_{5.6}$-Rho and (b) $Cu_{4.9}$-Rho samples.

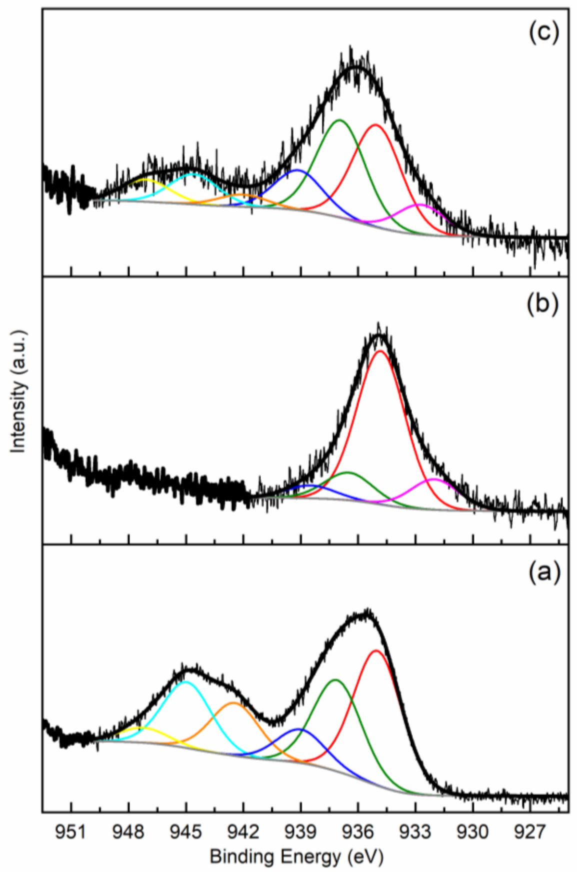

Figure S3. Deconvolution of the Cu $2p_{3/2}$ photoemission peaks of (a) CuO used as standard, (b) as prepared $Cu_{4.9}$-Rho and (c) heated $Cu_{4.9}$-Rho.

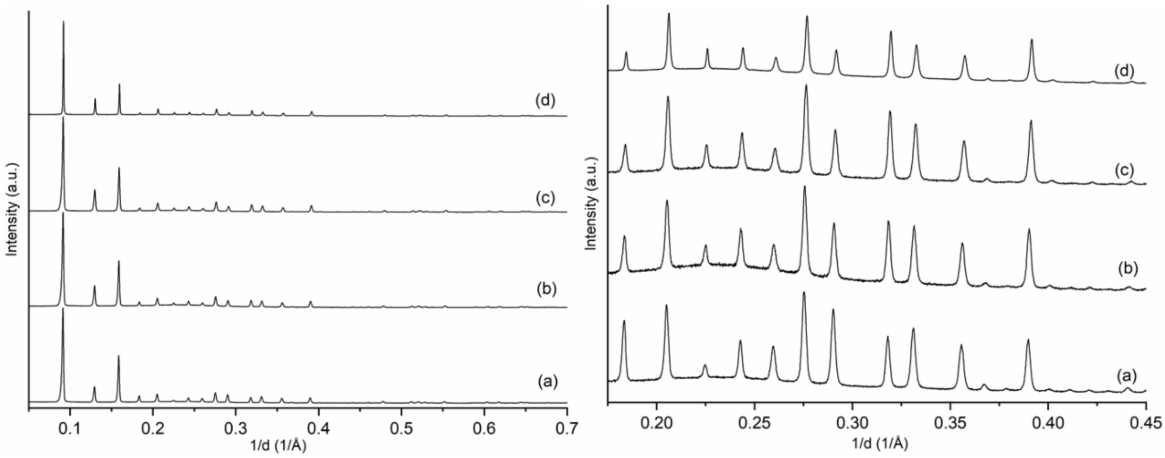

Figure S4. (Left) laboratory PXRD patterns of dehydrated (a) $Cu_{0.9}H_{8.0}$-Rho, (b) $Cu_{2.1}H_{5.6}$-Rho, (c) $Cu_{3.0}H_{3.8}$-Rho and (d) $Cu_{4.9}$-Rho. (Right) Magnified view of 1/d range from 0.175 1/Å to 0.45 1/Å.

Table S1. Crystallographic details of dehydrated samples.

	**$Cu_{0.9}H_{8.0}$-Rho**	**$Cu_{2.1}H_{5.6}$-Rho**	**$Cu_{3.0}H_{3.8}$-Rho**	**$Cu_{4.9}$-Rho**
**Unit cell**	$Cu_{0.9}H_{8.0}Al_{9.8}Si_{38.2}O_{96}$	$Cu_{2.1}H_{5.6}Al_{9.8}Si_{38.2}O_{96}$	$Cu_{3.0}H_{3.8}Al_{9.8}Si_{38.2}O_{96}$	$Cu_{4.9}Al_{9.8}Si_{38.2}O_{96}$
**Temperature/K**	298	298	298	298
**Space group**	$I\bar{4}3m$	$I\bar{4}3m$	$I\bar{4}3m$	$I\bar{4}3m$
**X-ray source**	Cu	Cu	Cu	Synchrotron
**Diffractometer**	Stoe	Stoe	Stoe	I-11
**Wavelength (Å)**	1.54056	1.54056	1.54056	0.826398
**a/ Å**	14.99471(17)	14.97435(17)	14.93520(16)	14.92589(8)
**Volume/Å^3**	3371.43(12)	3357.72(11)	3331.45(11)	3325.22(5)
**R_p**	0.0320	0.0280	0.0308	0.0183
**R_{wp}**	0.0423	0.0375	0.0420	0.0260
**χ^2**	2.509	1.837	2.583	11.52

	**$Cu_{1.0}Na_{7.8}$-Rho**	**$Cu_{3.4}Na_{3.0}$-Rho**	
**Unit cell**	$Cu_{1.0}Na_{7.8}Al_{9.8}Si_{38.2}O_{96}$	$Cu_{4.9}Al_{9.8}Si_{38.2}O_{96}$	$Cu_{3.0}Na_{3.9}Al_{9.8}Si_{38.2}O_{96}$
**Temperature/K**	298	298	
**Space group**	$I\bar{4}3m$	$Im\bar{3}m$	$I\bar{4}3m$
**X-ray source**	Synchrotron	Synchrotron	
**Diffractometer**	I-11	I-11	
**Wavelength (Å)**	0.826398	0.826398	
**a/ Å**	14.34496(6)	15.0324(18)	14.4052(9)
**Volume/Å^3**	2951.88(4)	3396.9(12)	2989.2(6)
**R_p**	0.0181	0.0278	
**R_{wp}**	0.0256	0.0393	
**χ^2**	8.504	35.45	

Table S2. Fractional atomic coordinates, occupancies, isotropic displacement parameters (in Å^2) and Cu-O distances (Å) for dehydrated samples.

**$Cu_{0.9}H_{8.0}$-Rho**	**x**	**y**	**z**	**Occup.**	**Multipl.**	**Uiso**

Si1	0.2499(5)	0.10441(31)	0.39880(32)	0.8	48	0.01876(29)
Al1	0.2499(5)	0.10441(31)	0.39880(32)	0.2	48	0.01876(29)
O1	0.17148(32)	0.17148(32)	0.369(88)	1.0	24	0.01876(29)
O2	0.16067(33)	0.16067(33)	0.6285(7)	1.0	24	0.01876(29)
O3	-0.0026(6)	0.21874(15)	0.37891(16)	1.0	48	0.01876(29)
Cu2	0.248(4)	0.248(4)	0.248(4)	0.1161(17)	8	0.05
**Distance**						
Cu2-O1	2.434(15)					
**$Cu_{2.1}H_{5.6}$-Rho**	**x**	**y**	**z**	**Occup.**	**Multipl.**	**Uiso**
Si1	0.2501(5)	0.1039(4)	0.3979(4)	0.8	48	0.01974(29)
Al1	0.2501(5)	0.1039(4)	0.3979(4)	0.2	48	0.01974(29)
O1	0.17310(27)	0.17310(27)	0.3651(6)	1.0	24	0.01974(29)
O2	0.15931(28)	0.15931(28)	0.6256(5)	1.0	24	0.01974(29)
O3	-0.0001(5)	0.21998(15)	0.37902(16)	1.0	48	0.01974(29)
Cu2	0.2489(22)	0.2489(22)	0.2489(22)	0.2635(19)	8	0.05
**Distance**						
Cu2-O1	2.368(10)					
**$Cu_{3.0}H_{3.8}$-Rho**	**x**	**y**	**z**	**Occup.**	**Multipl.**	**Uiso**
Si1	0.2506(5)	0.1037(4)	0.3976(4)	0.8	48	0.02121(29)
Al1	0.2506(5)	0.1037(4)	0.3976(4)	0.2	48	0.02121(29)
O1	0.17362(25)	0.17362(25)	0.3614(5)	1.0	24	0.02121(29)
O2	0.15869(28)	0.15869(28)	0.6240(4)	1.0	24	0.02121(29)
O3	-0.0007(6)	0.22028(17)	0.37794(16)	1.0	48	0.02121(29)
Cu1	0.6324(26)	0.046(5)	0.014(11)	0.0108(5)	48	0.05
Cu2	0.2492(19)	0.2492(19)	0.2492(19)	0.3171(19)	8	0.05
**Distance**						
Cu1-O3	2.615					
Cu2-O1	2.314(9)					
**$Cu_{4.9}$-Rho**	**x**	**y**	**z**	**Occup.**	**Multipl.**	**Uiso**
Si1	0.25333(25)	0.10453(18)	0.39666(17)	0.8	48	0.02197(13)
Al1	0.25333(25)	0.10453(18)	0.39666(17)	0.2	48	0.02197(13)
O1	0.15848(19)	0.15848(19)	0.37753(27)	1.0	24	0.02197(13)
O2	0.17487(18)	0.17487(18)	0.6363(4)	1.0	24	0.02197(13)
O3	0.0032(4)	0.22202(12)	0.37986(11)	1.0	48	0.02197(13)
Cu1	0.6475(5)	0.0839(7)	-0.0274(9)	0.0423(4)	48	0.05
Cu2	0.2501(7)	0.2501(7)	0.2501(7)	0.3592(12)	8	0.05
**Distance**						
Cu1-O1	2.282(12)					
Cu1-O3	2.133(10)					
Cu2-O2	2.323(4)					
**$Cu_{1.0}Na_{7.8}$-Rho**	**x**	**y**	**z**	**Occup.**	**Multipl.**	**Uiso**
Si1	0.27371(5)	0.12433(6)	0.42459(6)	0.8	48	0.01257(19)
Al1	0.27371(5)	0.12433(6)	0.42459(6)	0.2	48	0.01257(19)
O1	0.03721(10)	0.21160(10)	0.38518(11)	1.0	48	0.01257(19)
O2	0.21997(12)	0.21997(12)	0.40195(19)	1.0	24	0.01257(19)
O3	0.12142(13)	0.12142(13)	0.62397(16)	1.0	24	0.01257(19)
Na1	0.3910(4)	0.02788(30)	0.02788(30)	0.2474(15)	24	0.05
Cu1	0.2827(4)	0.2827(4)	0.2827(4)	0.1262(16)	8	0.05
Na2	0.30536	0.30536	0.30536	0.21077	8	0.05
**Distance**						
Cu1-O2	2.1322(1)					
**$Cu_{3.4}Na_{3.0}$-Rho**						
**$Cu_{4.9}$ -Rho (phase 1)**	**x**	**y**	**z**	**Occup.**	**Multipl.**	**Uiso**
Si1	0.25	0.1083(12)	0.3917(12)	0.8	48	0.003(4)

Al1	0.25	0.1083(12)	0.3917(12)	0.2	48	0.003(4)
O1	0.0	0.2575(32)	0.4047(24)	1.0	48	0.003(4)
O2	0.1635(21)	0.1635(21)	0.3789(32)	1.0	48	0.003(4)
Cu2	0.243(9)	0.243(9)	0.243(9)	0.289(19)	16	0.06
**Distance**						
Cu2-O2	2.651					
**$Cu_{3.0}Na_{3.9}$-Rho (phase 2)**	**x**	**y**	**z**	**Occup.**	**Multipl.**	**Uiso**
Si1	0.2736(5)	0.1232(6)	0.4235(6)	0.8	48	0.0120(13)
Al1	0.2736(5)	0.1232(6)	0.4235(6)	0.2	48	0.0120(13)
O1	0.0350(11)	0.2127(10)	0.3834(11)	1.0	48	0.0120(13)
O2	0.2190(13)	0.2190(13)	0.3992(19)	1.0	24	0.0120(13)
O3	0.1214(14)	0.1214(14)	0.6258(17)	1.0	24	0.0120(13)
Na3	0.400(5)	0.022(4)	0.022(4)	0.166(13)	24	0.01
Cu1	0.2785(11)	0.2785(11)	0.2785(11)	0.373(13)	8	0.001
**Distance**						
Cu1-O2	2.120(29)					

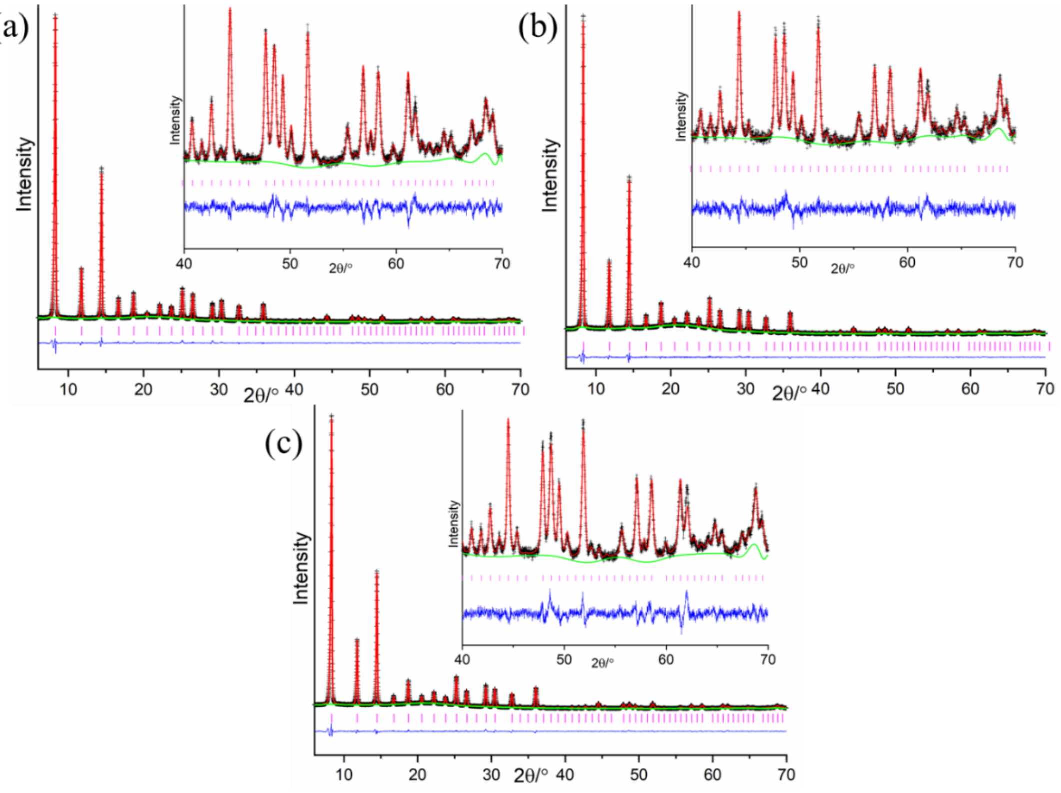

Figure S5. Rietveld plots of PXRD profiles (λ = 1.54056 Å, T = 298 K) of dehydrated (a) $Cu_{0.9}H_{8.0}$-Rho, (b) $Cu_{2.1}H_{5.6}$-Rho and (c) $Cu_{3.0}H_{3.8}$-Rho. (Observed – black, calculated – red, difference – blue, phase – pink and background – green).

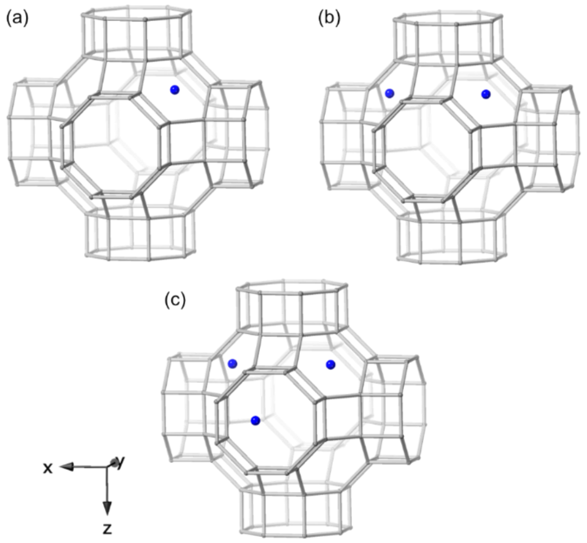

Figure S6. Structural representation of cation positions in zeolite (a) $Cu_{0.9}H_{8.0}$-Rho, (b) $Cu_{2.1}H_{5.6}$-Rho and (c) $Cu_{3.0}H_{3.8}$-Rho. Cu^{2+} cations = blue spheres. Framework O atoms are omitted for clarity and T–T linkages are represented by grey rods.

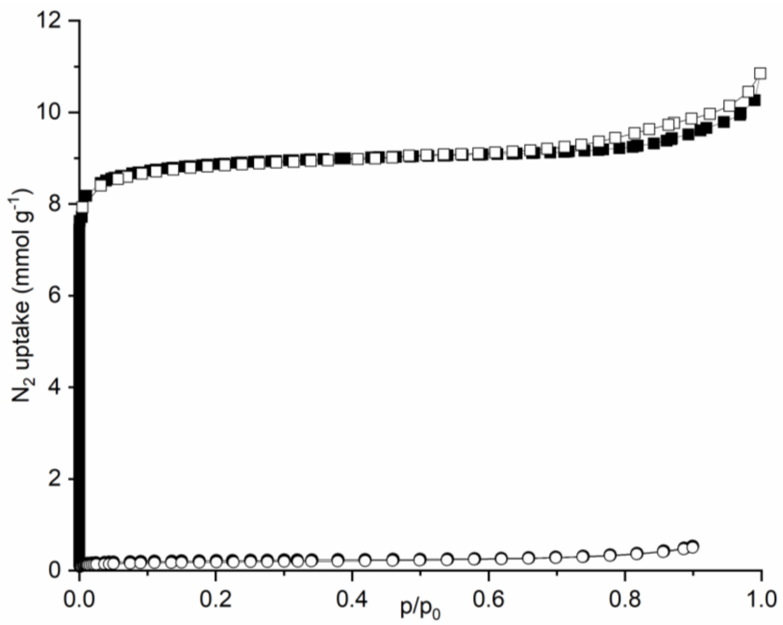

Figure S7. N_2 adsorption at 77 K on $Cu_{4.9}$-Rho (squares) and $Na_{9.8}$-Rho (circles) samples. Adsorption = closed symbols; desorption = open symbols.

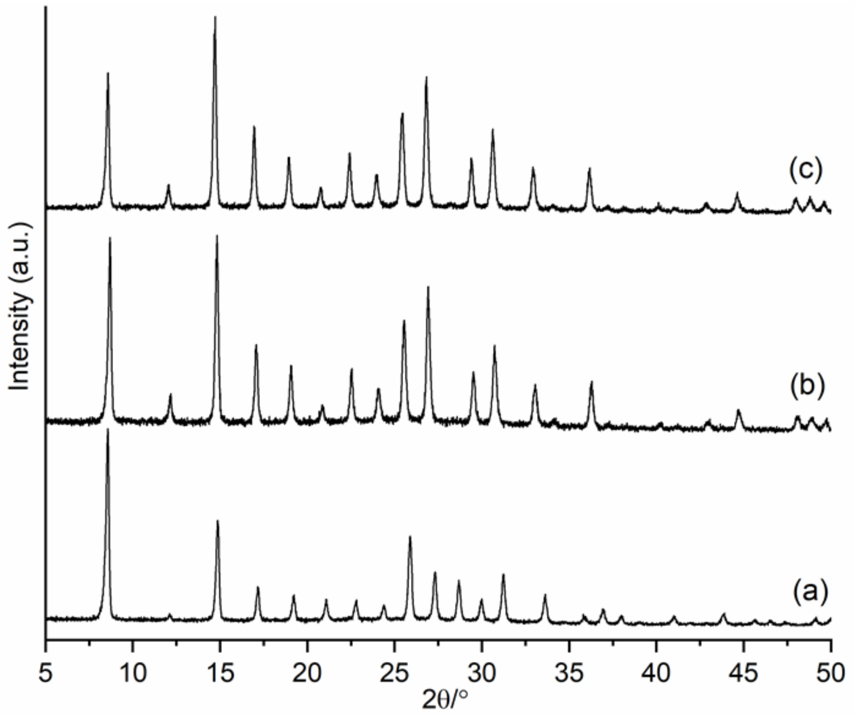

Figure S8. XRD patterns of hydrated (a) $Na_{9.8}$-Rho, (b) $Cu_{1.0}Na_{7.8}$-Rho and (c) $Cu_{3.4}Na_{3.0}$-Rho.

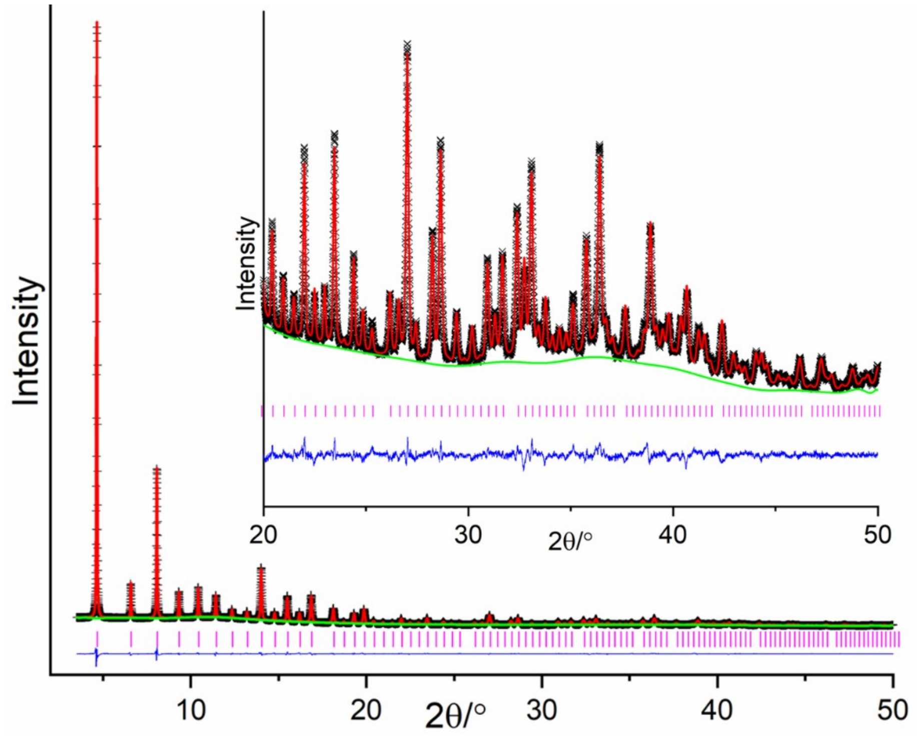

Figure S9. Rietveld plot of synchrotron PXRD profile (λ = 0.8263980 Å, T = 298 K) of dehydrated $Cu_{1.0}Na_{7.8}$-Rho. (Observed – black, calculated – red, difference – blue, phase – pink and background – green).

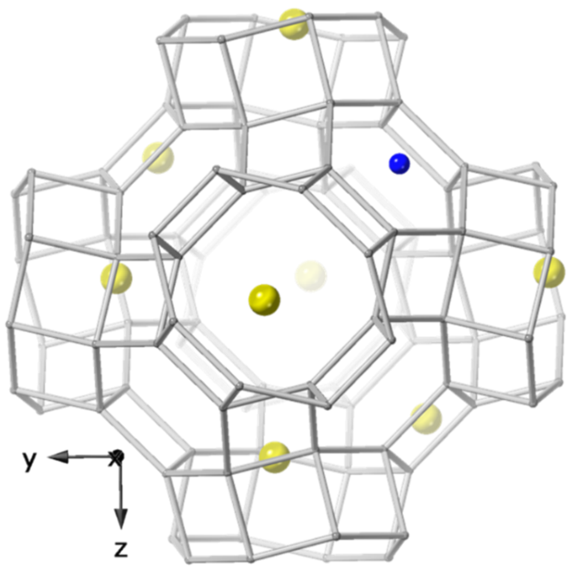

Figure S10. Generalised model of the structure of zeolite $Cu_{1.0}Na_{7.8}$-Rho obtained from synchrotron data. The Cu^{2+} cations = blue spheres, Na^{+} cations = yellow spheres. Framework O atoms are omitted for clarity and T–T linkages are represented by grey rods.

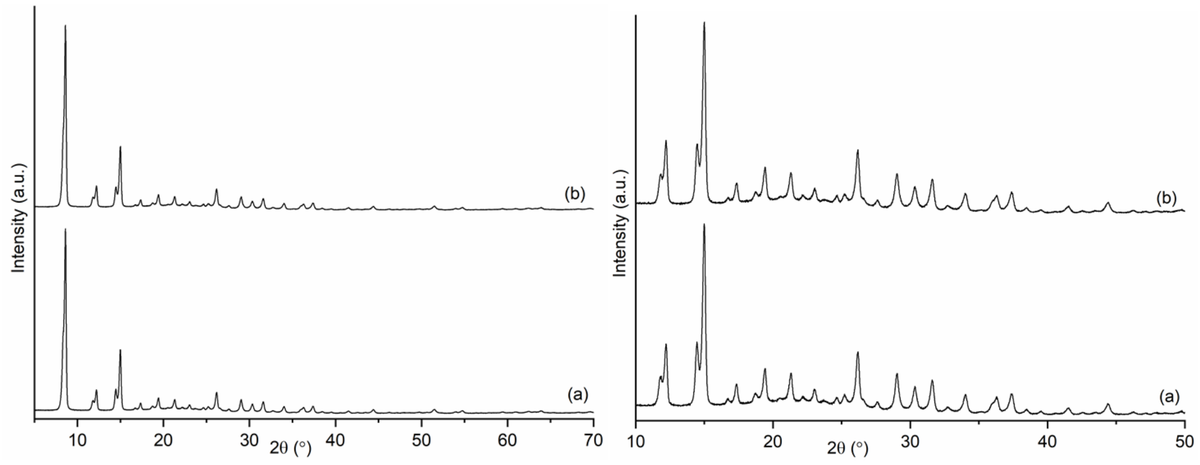

Figure S11. (Left) XRD of $Cu_{3.4}Na_{3.0}$-Rho dehydrated at 623 K for (a) 10 hours and (b) 20 hours. (Right) Magnified view of 2θ range from 10º to 50º.

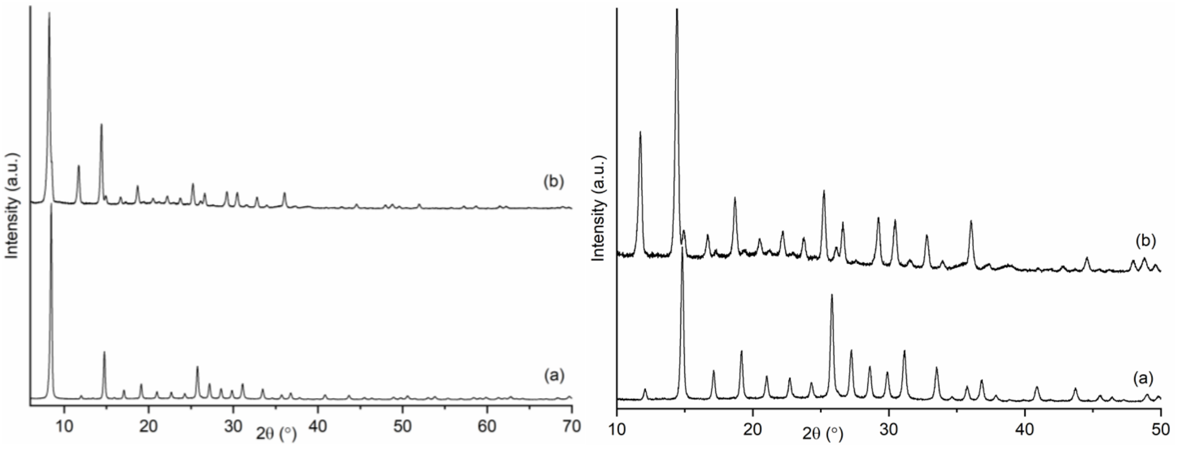

Figure S12. (Left) XRD patterns of dehydrated (a) $Na_{9.8}$-Rho and (b) $Cu_{4.3}Na_{1.0}$-Rho to indicate the position of peaks for the acentric phase. (Right) Magnified view of 2θ range from 10º to 50º.

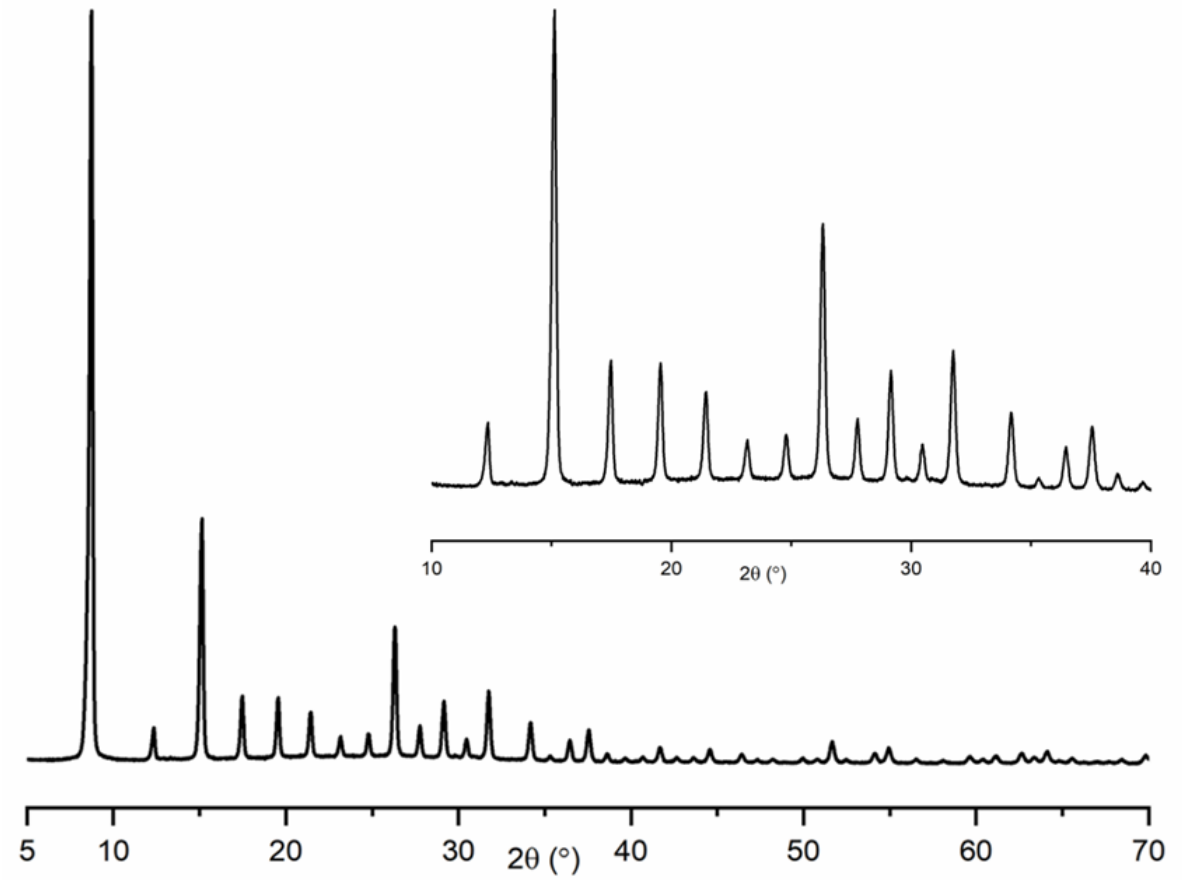

Figure S13. XRD pattern of dehydrated $Na_{4.5}H_{5.3}$-Rho (a = 14.3447(2) Å) with a magnified view of 2θ range from 10 – 40º.

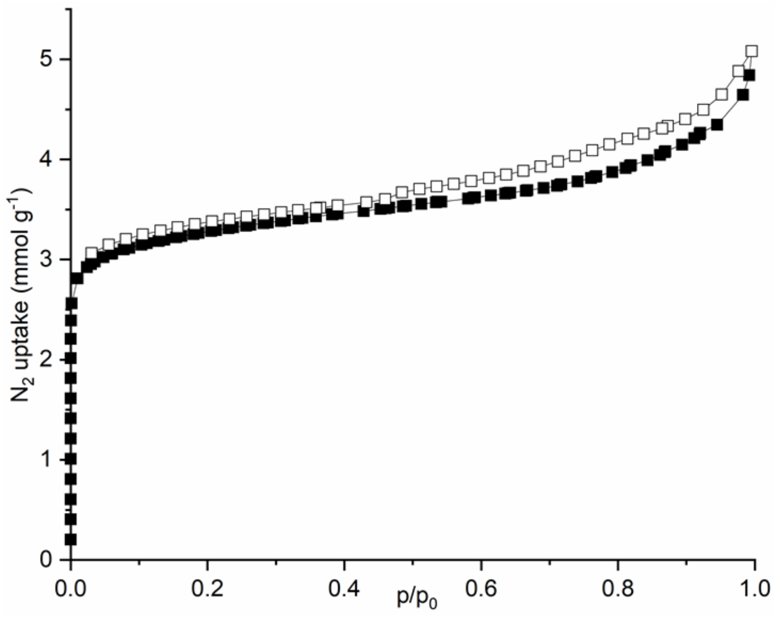

Figure S14. N_2 adsorption at 77 K on $Cu_{3.4}Na_{3.0}$-Rho sample. Adsorption = closed symbols; desorption = open symbols.

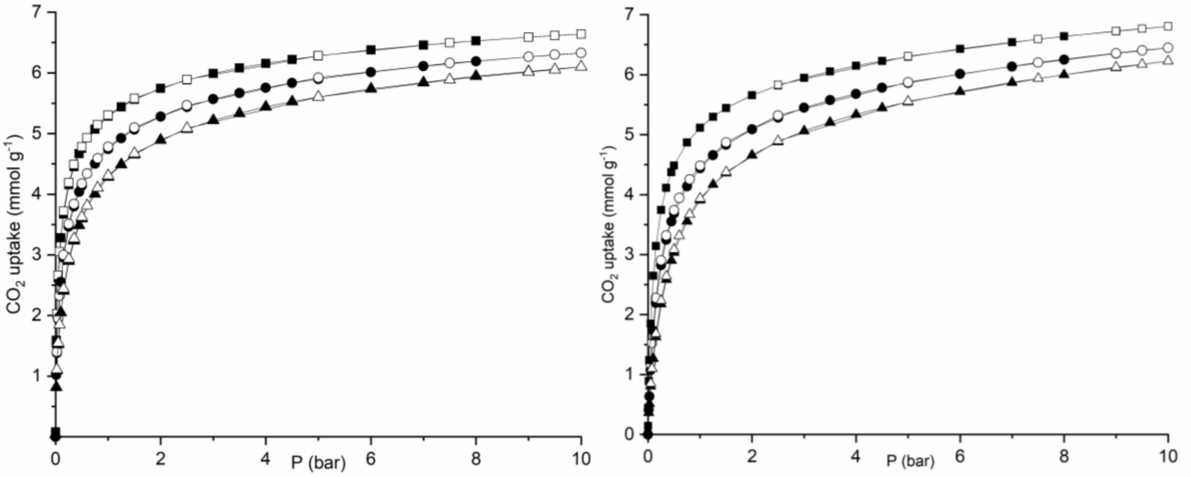

Figure S15. High pressure CO_2 isotherms on $Cu_{3.0}Na_{3.9}$-Rho (left) and $Cu_{4.9}$-Rho (right) at 283 K (■), 298 K (●) and 313 (▲). Adsorption, closed symbols; desorption, open symbols.

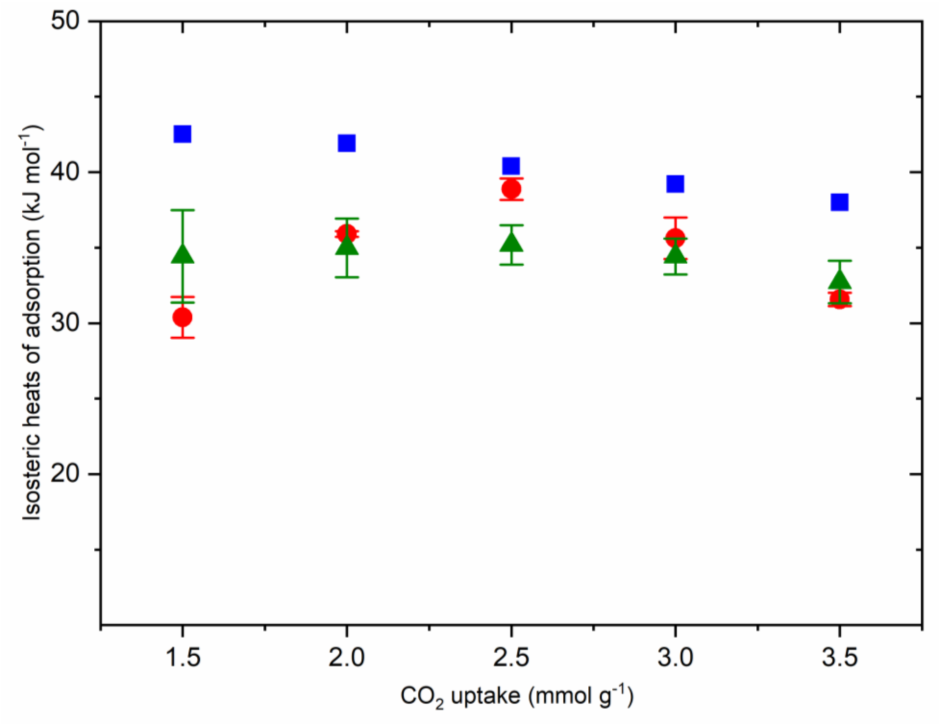

Figure S16. Isosteric heats of adsorption measured over the range of 1.5–3.5 mmol g^{-1} for $Na_{9.8}$-Rho (blue), $Cu_{3.0}Na_{3.9}$-Rho (red) and $Cu_{4.9}$-Rho (green). Data for $Na_{9.8}$-Rho were previously published in ref.[2]

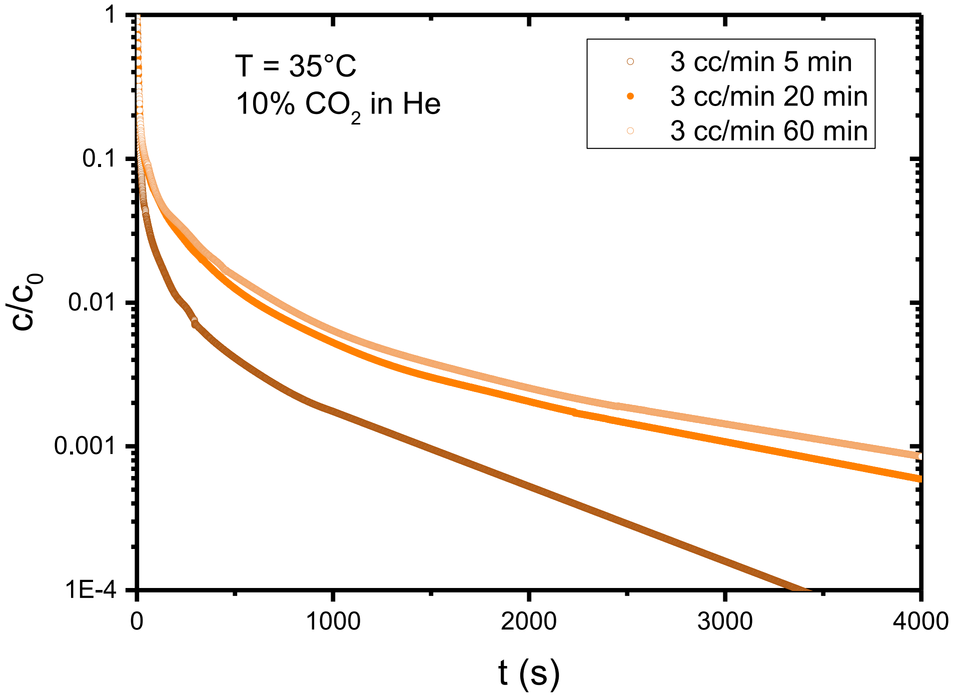

Figure S17. The ZLC measurements at 308 K for $Na_{9.8}$-Rho (m = 4.0 mg). Due to the slow kinetics, partial loading experiments were carried out to obtain the diffusivity.

The data shown is deconvoluted so as to remove contributions to the desorption profile which are inherent to the experimental setup.[3]

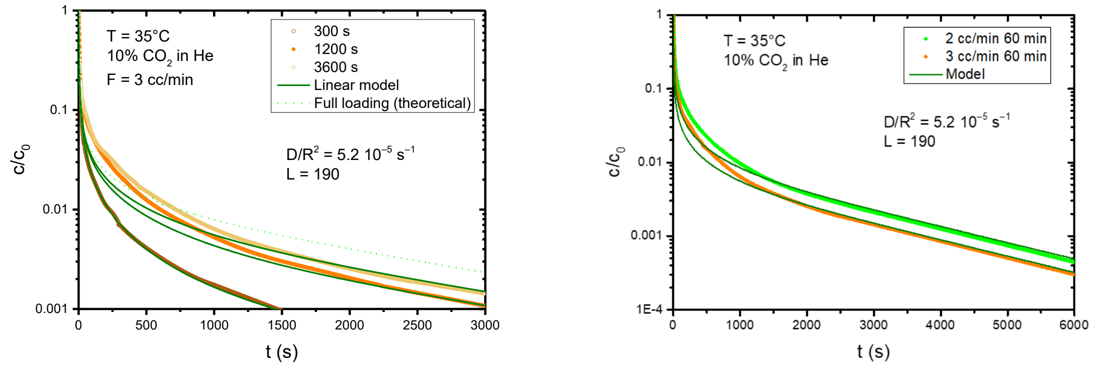

Figure S18. Deconvoluted ZLC data at 308 K on Na9.8-Rho sample in Ft plot and versus time with model fits using the analytical solution for linear isotherms, $D/R^2 = 5.2 \times 10^{-5}$ s^{-1}.

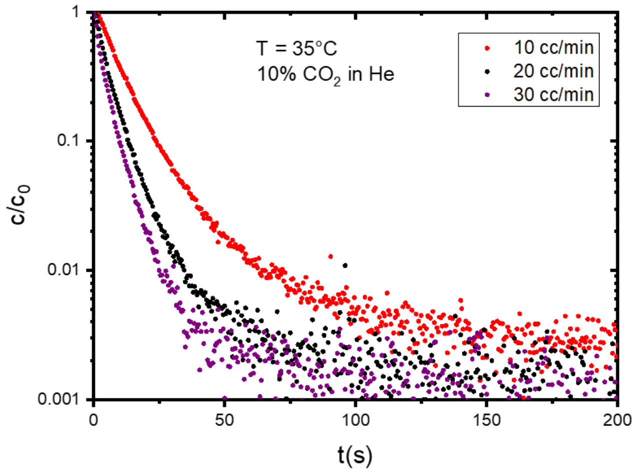

Figure S19. The ZLC measurements at 308 K for Cu4.9-Rho (m = 5.79 mg).

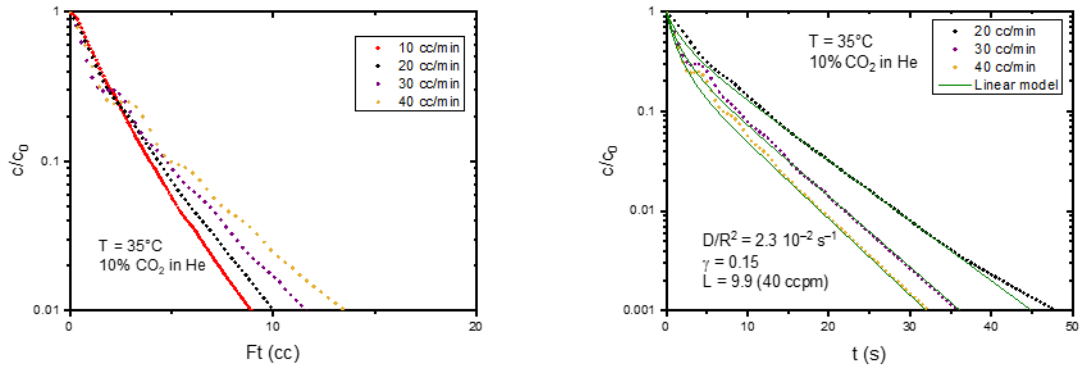

Figure S20. Deconvoluted ZLC data at 308 K on $Cu_{4.9}$-Rho sample in Ft plot and versus time with model fits using the analytical solution for linear isotherms. $D/R^2 = 2.3\ 10^{-2}\ s^{-1}$

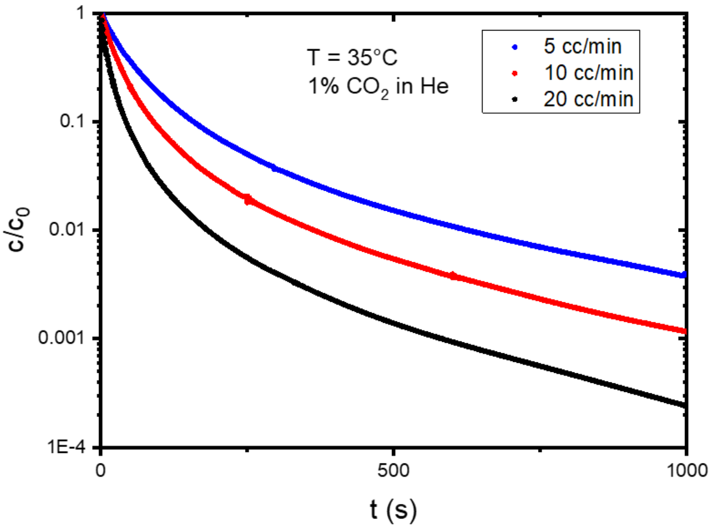

Figure S21. The ZLC measurements at 308 K for $Cu_{3.4}Na_{3.0}$-Rho (m = 6.78 mg).

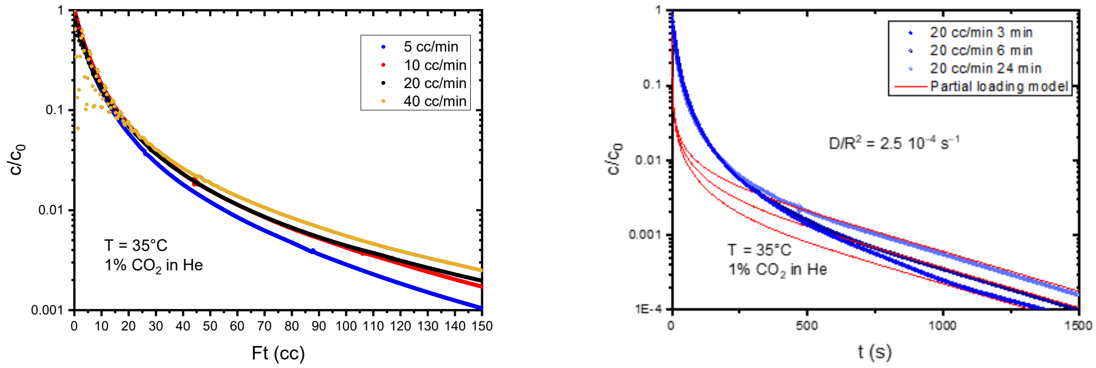

Figure S22. Deconvoluted ZLC data at 308 K on $Cu_{3.4}Na_{3.0}$-Rho sample in Ft plot and versus time. The Ft plot shows overlapping curves initially, down to $c/c_0 = 0.1$, below which the curves diverge, indicating fast kinetics above this concentration and kinetic limitations below. Partial loading experiments were carried out to obtain the diffusivity at low concentrations ($p_{CO2} < 100$ Pa) with model fits using the analytical solution for linear isotherms. $D/R^2 = 2.5\ 10^{-4}\ s^{-1}$

References

[1] T. Chatelain, J. Patarin, E. Fousson, M. Soulard, J. L. Guth, P. Schulz, *Microporous Mater.* **1995**, *4*, 231–238.

[2] M. M. Lozinska, J. P. S. Mowat, P. A. Wright, S. P. Thompson, J. L. Jorda, M. Palomino, S. Valencia, F. Rey, *Chem. Mater.* **2014**, *26*, 2052–2061.

[3] M. Verbraeken, A. Centineo, L. Canobbio, S. Brandani, *Adsorption* **2021**, *27*, 129–145.
